# Supplementary material for: YouTube and the implementation and discontinuation of the oral contraceptive pill: A mixed-method content analysis
Source: PLoS One. 2024 May 24;19(5):e0302316. doi: 10.1371/journal.pone.0302316 (PMC11125465; doi:10.1371/journal.pone.0302316)
Supplement: S1 Table — (DOCX) [file pone.0302316.s001.docx]

**S1 Table. German Search Terms**

| Search Strategy |
| --- |
| “Pille absetzen” |
| “Pille absetzen Erfahrungen” |
| “Absetzen der Pille” |
| “Absetzen der Pille Erfahrungen” |
