# Supplementary material for: YouTube and the implementation and discontinuation of the oral contraceptive pill: A mixed-method content analysis
Source: PLoS One. 2024 May 24;19(5):e0302316. doi: 10.1371/journal.pone.0302316 (PMC11125465; doi:10.1371/journal.pone.0302316)
Supplement: S2 Table — (DOCX) [file pone.0302316.s002.docx]

**S3 Table. Qualitative coding scheme**

| Main category | Subcategory | Rule | Anchor example |
| --- | --- | --- | --- |
| General facts  Definition:  General facts and experiences of YouTubers about taking and stopping the contraceptive pill. | Time of video recording after discontinuing the contraceptive pill | Statements of the YouTuber about the period of discontinuation | „As I said, I stopped taking the pill two years ago.“ (P002, line 7) |
|  | Age at first dose | Mentioning of the age at the first dose | „Namely I was there (1) 15 […].“ (P001, line 25) |
|  | Age at discontinuation | Naming the age at discontinuing the pill/ *results from the age at first dose plus persistence with the contraceptive pill* | „[...] and continued to take until I'm just now 22 [...]. (P001, line 43) |
|  | Length of implementation | Mentioning the period of taking the contraceptive pill. | „So, I was on the pill for seven years straight [...] (P001, line 44 et seq.) |
|  | Reasons for taking the contraceptive pill | Statements justifying the use of the pill | „[...] I actually really took it at the time because I had bad skin [...]. “ (P002, line 17 et seq.) |
|  | Reasons for discontinuation | Statements on the part of the YouTubers that justify the discontinuation or are related to the process of discontinuation. | „[...] the pill is really shit for the body in the long run.“ (P001, line 96 et seq.) |
|  | Second discontinuation | Mentioning a second discontinuation of the contraceptive pill. | “And that was it with me and this contraceptive method. After that I never took the pill again.” (P018, line 75 et seq.) |
|  | Type of contraceptive pill | Naming of a medication. Naming of the corresponding generation of the contraceptive pill. Change of the type of the contraceptive pill during the intake. |  |
|  | Switch between contraceptive pills | Mention of switch from one contraceptive pill to another. | “He prescribed me another pill and from that point on I only had problems with the pill. All the side effects that I had before were much worse and I only really noticed them once.” (P010, line 46 et seq.) |
|  | Current contraceptive method | Mention of the current contraceptive method | „And I then decided on a copper IUD“ (P002, line 30 et seq.) |
|  | Conclusion of contraceptive pill discontinuation. | Final summary on stopping the contraceptive pill | „I am doing very very well, that is already the conclusion. I have absolutely no regrets about stopping the pill [...].” (P002, line 13 et seq.) |
| Desirable and undesirable effects of the contraceptive pill during implementation.  Definition:  Statements about adverse effects (side effects) as well as desired (positive) effects of the pill while taking it. | Adverse effects (negative side effects) | Statements on undesirable effects, symptoms during implementation. | „I had a headache every day and it just wouldn't go away“. (P001, line 58 et seq.) |
|  | Desired effects (positive side effects)/improvements | Statements about desired, positive effects or improvements during implementation. | „And my skin actually got better then [...].“ (P002, line 21 et seq.) |
| Physiological and psychological changes after discontinuation of the pill.  Definition:  Statements regarding both positive and negative physiological and psychological changes associated with discontinuation of the pill. Additional consideration is given to the timing and extent of these changes. | Physiological | Statements on physiological changes | „But I got a very very blemished skin […]“ (V002, line 117) |
|  | Psychological | Statements about psychological changes | „Now I am much more relaxed […].“ (P002, line 252) |
|  | *Time at which the change occurred* |  | „So hair loss, that was also so three months or so after I stopped taking the pill“ (P002, line 205 et seq.) |
|  | *Length of the stop of the change* |  | „[…]a very short time times that I have noticed that somehow two weeks times strongly or three […].“ (P002, line 213 et seq.) |
| *Attitudes* of YouTubers toward physiological and psychological changes.  Definition:  Reactions and evaluative statements of YouTubers regarding the changes as well as indirect or direct recommendations that are intended to motivate them to act in a certain way. | *Change assessment* | Personal evaluation and opinion towards the occurring changes. | „Skin is not the most important thing in life [...]." (P002, line 190)  "Other people don't see it so bad at all.“ (P002, line 194 et seq.) |
|  | *Reaction to changes* | Personal reaction (emotions, thoughts, feelings, actions) regarding the changes that occur. | „[…] I felt very uncomfortable […]“ (P002, line 146 et seq.) |
| Attitude toward the pill, hormonal contraception.  Definition:  Expressions of opinion, thoughts, and feelings of YouTubers toward the pill, hormonal contraception. | Evaluation of the contraceptive pill | Personal opinion and assessment of the pill as well as hormonal contraception overall. Both positively and negatively evaluated aspects of the pill/ hormonal contraception *Thoughts, feelings towards the pill.* | „[…] Advantages for the pill (1) for me (1) that you do not have pain […].“ (P002, line 272) |
| Experiences with gynecologists  Definition:  Discussions with gynecologists about the choice or change of contraceptive method. | Advice from the gynecologist before taking the pill | Testimonies of counseling sessions regarding the use of the pill, the choice of the first contraceptive method | “My gynecologist was fortunately also so understanding […].” (P119, line 56) |
|  | Advice from: the gynecologist regarding discontinuation of the contraceptive pill | Statements from counseling sessions regarding discontinuing the pill, changing contraceptive methods | „He then recommended the hormonal IUD to me […].“ (V001, line 102 et seq.) |
| Source of information |  | Description of sources, that were used to gain information regarding the (discontinuation of the) contraceptive pill | “I learned a little bit about it via the Internet and through my friends, that they no longer take the pill and what negative sides the pill brings with it, and for me that was always in the back of my mind […]” (P007, line 47 et seq.) |
