# Supplementary material for: YouTube and the implementation and discontinuation of the oral contraceptive pill: A mixed-method content analysis
Source: PLoS One. 2024 May 24;19(5):e0302316. doi: 10.1371/journal.pone.0302316 (PMC11125465; doi:10.1371/journal.pone.0302316)
Supplement: S3 Table — (DOCX) [file pone.0302316.s003.docx]

**S4 Table. Video descriptives, detail** (N = 175)

| Characteristics | Min | Max | Median | Mean | Standard deviation | Sum |
| --- | --- | --- | --- | --- | --- | --- |
| Dates posted | 2014 | 2023 | 2019 | 2018.8 | 2.0 | 2020 (Modus) |
| Views | 13.0 | 1,586,690.0 | 8,772.00 | 49,550.3 | 152,129.2 | 8,621,746.0 |
| Length (Minutes) | 5.4 | 57.0 | 14.2 | 14.8 | 6.1 | 25,955.0 |
| Likes | 0.0 | 70,315.0 | 134.0 | 1,541.0 | 5,868.6 | 266,600.0 |
| Comments | 0.0 | 5,467.0 | 34.0 | 166.4 | 484.9 | 27,793.0 |
