# Supplementary material for: YouTube and the implementation and discontinuation of the oral contraceptive pill: A mixed-method content analysis
Source: PLoS One. 2024 May 24;19(5):e0302316. doi: 10.1371/journal.pone.0302316 (PMC11125465; doi:10.1371/journal.pone.0302316)
Supplement: S1 File — (PDF) [file pone.0302316.s004.pdf]

S2 File. Translated codebook for the quantitative content analysis.

General notes and encoding instructions:

- The coding material is the videos themselves, as well as the title and text below the video.
- The quantitative content analysis will be conducted on videos by YouTubers who have personal experiences with stopping the pill and who discuss them in the videos. Video excerpts that do not contain any information about personal experiences after stopping the pill are not considered.
- We distinguish between category dimensions and categories. Category dimensions are groups of logically related categories.
    - The category dimensions can be combined from several dichotomous variables (1= mentioned, 0= not mentioned) in the later data analysis.
    - Are summarized in the evaluation as potential multiple answers in the variables.
    - Only the individual categories are listed in the category system. Category dimensions are not merged until later. However, they can be recognized by the same ordinal numbers (1.4.1, 1.4.2, or 1.4.1.1, 1.4.1.2).
- If STRING is provided to operationalize the variables, meaningful summary variables will be created from the text responses in the data analysis.
- If categories are not mentioned in the whole video, they are coded with '. In this way, the data can be passed directly to the statistics program as MISSING.
- The coding material is first coded in MAXQDA and then transferred to Excel. Further data analysis and presentation is done in STATA.

Category system

1. Formal categories

These categories capture the formal characteristics of YouTube videos.

Nr.	Category/Code	Values and values labels
1.1	Video identification number	V001
1.2	Individual identification number	P001
1.3	Channel identification number	C001
1.4	Date of upload	01.01.2021
1.5	Video title	STRING
1.6	Number of views	NUMERIC
1.7	Video length	00:05:00-…
1.8	Number of likes	NUMERIC
1.09	Number of comments	NUMERIC
1.10	Disclaimer	0 = mentioned, 1 = not mentioned

2. General content categories

These content categories are intended to provide general facts about YouTubers experiences, taking the pill and coming off the pill.

Nr.	Category/Code	Values and values labels
2.1	Discontinuation 1	NUMERIC
2.2	Discontinuation 2	NUMERIC
2.3	Discontinuation 3	NUMERIC
2.4	Length of pill intake (years)	NUMERIC
2.4.1	Begin of pill intake (age)	NUMERIC
2.4.2	Discontinuation of pill (age)	NUMERIC
2.5	Sort of pill	0 = mentioned, 1 = not mentioned
2.5.1	Name of pill I	STRING
2.5.1.1	Generation of pill I	1-4
2.5.2	Name of pill II	STRING
2.5.2.1	Generation of pill II	1-4
2.5.3	Name of pill III	STRING
2.5.3.1	Generation of pill III	1-4
2.6	Second discontinuation	0 = mentioned, 1 = not mentioned
2.7	Switch of pill during intake	0 = mentioned, 1 = not mentioned
2.8	Final conclusion regarding discontinuation	0 = negative, 1 = indifferent, 2 = positive
2.9	Reasons for intake	STRING
2.9.1	Reasons for intake skin	0 = mentioned, 1 = not mentioned
2.9.2	Reasons for intake painful bleeding	0 = mentioned, 1 = not mentioned

2.9.3	Reasons for intake skin contraception	0 = mentioned, 1 = not mentioned
2.9.4	Reasons for intake skin partner	0 = mentioned, 1 = not mentioned
2.9.5	Reasons for intake skin gynaecologist	0 = mentioned, 1 = not mentioned
2.9.6	Reasons for intake skin friends	0 = mentioned, 1 = not mentioned
2.9.7	Reasons for intake skin irregular bleeding	0 = mentioned, 1 = not mentioned
2.10	Reasons for discontinuation	STRING
2.10.1	Reasons for discontinuation side effects	0 = mentioned, 1 = not mentioned
2.10.2	Reasons for discontinuation fear of side effects	0 = mentioned, 1 = not mentioned
2.11	Current contraceptive method	STRING
2.12	Differentiation Menstruation and abortion bleeding	0 = mentioned, 1 = not mentioned
2.13	Relevant supply groups	STRING
2.14	Recognition of symptoms after discontinuation	0 = mentioned, 1 = not mentioned

3. Content data pill intake

Nr.	Category/Code	Values and value labels
3.1	Improvements during pill intake	STRING
3.2	Physiological side effects	/
3.2.1	Hair loss	0 = mentioned, 1 = not mentioned
3.2.2	Oily hair	0 = mentioned, 1 = not mentioned
3.2.3	Skin impurities	0 = mentioned, 1 = not mentioned
3.2.4	Dry skin	0 = mentioned, 1 = not mentioned
3.2.5	Cycle discontinuation	0 = mentioned, 1 = not mentioned
3.2.6	Absence bleeding	0 = mentioned, 1 = not mentioned
3.2.7	Painful bleeding	0 = mentioned, 1 = not mentioned
3.2.8	Spotting	0 = mentioned, 1 = not mentioned
3.2.9	Weight gain	0 = mentioned, 1 = not mentioned
3.2.10	Hydrocephalus	0 = mentioned, 1 = not mentioned
3.2.11	Weight gain breast	0 = mentioned, 1 = not mentioned
3.2.12	Headache	0 = mentioned, 1 = not mentioned
3.2.13	Migraine	0 = mentioned, 1 = not mentioned
3.2.14	Blooding	0 = mentioned, 1 = not mentioned
3.2.15	Breast pain	0 = mentioned, 1 = not mentioned
3.2.16	Vaginal dryness	0 = mentioned, 1 = not mentioned
3.2.17	Painful legs	0 = mentioned, 1 = not mentioned
3.2.18	Decreased feel of saturation	0 = mentioned, 1 = not mentioned
3.2.19	Exhaustion	0 = mentioned, 1 = not mentioned
3.2.20	Other	STRING
3.3	Psychological side effects	/
3.3.1	Deuteration Libido	0 = mentioned, 1 = not mentioned
3.3.2	Depressive moodiness	0 = mentioned, 1 = not mentioned
3.3.3	Depression	0 = mentioned, 1 = not mentioned
3.3.4	Mood swings	0 = mentioned, 1 = not mentioned
3.3.5	Other	STRING

4. Content data pill discontinuation

Nr.	Category/Code	Values and value labels
4.1	Physiological changes	/
4.1.1	Hair loss	0 = mentioned, 1 = not mentioned
4.1.1.1	Hair loss start (month)	NUMERIC
4.1.1.2	Hair loss end (month)	NUMERIC
4.1.2	Oily hair	0 = mentioned, 1 = not mentioned
4.1.2.1	Oily hair start (month)	NUMERIC
4.1.2.2	Oily hair end (month)	NUMERIC
4.1.3	Skin impurities	0 = mentioned, 1 = not mentioned
4.1.3.1	Skin impurities begin (month)	NUMERIC
4.1.3.2	Skin impurities end (month)	NUMERIC
4.1.4	Oily skin	0 = mentioned, 1 = not mentioned
4.1.4.1	Oily skin begin (month)	NUMERIC
4.1.4.2	Oily skin end (month)	NUMERIC
4.1.5	Hormonal acne	0 = mentioned, 1 = not mentioned
4.1.5.1	Hormonal acne begin (month)	NUMERIC
4.1.5.2	Hormonal acne end (month)	NUMERIC
4.1.6	First period (month)	NUMERIC
4.1.7	Cycle discontinuities	0 = mentioned, 1 = not mentioned
4.1.7.1	Cycle discontinuities begin (month)	NUMERIC
4.1.7.2	Cycle discontinuities end (month)	NUMERIC
4.1.8	Strong bleeding	0 = mentioned, 1 = not mentioned
4.1.8.1	Strong bleeding begin (month)	NUMERIC
4.1.8.2	Strong bleeding end (month)	NUMERIC
4.1.9	Weight gain	0 = mentioned, 1 = not mentioned
4.1.9.1	Weight gain begin (month)	NUMERIC
4.1.9.2	Weight gain end (month)	NUMERIC
4.1.10	Weight loss	0 = mentioned, 1 = not mentioned
4.1.10.1	Weight loss begin (month)	NUMERIC
4.1.10.2	Weight loss end (month)	NUMERIC
4.1.11	Weight loss breasts	0 = mentioned, 1 = not mentioned
4.1.11.1	Weight loss breasts begin (month)	NUMERIC
4.1.11.2	Weight loss breasts end (month)	NUMERIC
4.1.12	Breast pain	0 = mentioned, 1 = not mentioned
4.1.12.1	Breast pain begin (month)	NUMERIC
4.1.12.2	Breas pain end (month)	NUMERIC
4.1.13	Headache	0 = mentioned, 1 = not mentioned
4.1.13.1	Headache begin (month)	NUMERIC
4.1.13.2	Headache end (month)	NUMERIC
4.1.14	Migraine	0 = mentioned, 1 = not mentioned
4.1.14.1	Migraine begin (month)	NUMERIC
4.1.14.2	Migraine end (month)	NUMERIC
4.1.15	Nausea	0 = mentioned, 1 = not mentioned
4.1.15.1	Nausea begin (month)	NUMERIC
4.1.15.2	Nausea end (month)	NUMERIC
4.1.16	Blooding	0 = mentioned, 1 = not mentioned
4.1.16.1	Blooding begin (month)	NUMERIC

4.1.16.2	Blooding end (month)	NUMERIC
4.1.17	Hair growth	0 = mentioned, 1 = not mentioned
4.1.17.1	Hair growth (month)	NUMERIC
4.1.17.2	Hair growth (month)	NUMERIC
4.1.18	Sweating	0 = mentioned, 1 = not mentioned
4.1.18.1	Sweating begin (month)	NUMERIC
4.1.18.2	Sweating end (month)	NUMERIC
4.1.19	Other	STRING
4.1.20	Improvements	STRING
4.2	Psychological changes	/
4.2.1	Depressive moodiness	0 = mentioned, 1 = not mentioned
4.2.1.1	Depressive moodiness start (month)	NUMERIC
4.2.1.2	Depressive moodiness end (month)	NUMERIC
4.2.2	Depression	0 = mentioned, 1 = not mentioned
4.2.2.1	Depression start (month)	NUMERIC
4.2.2.2	Depression end (month)	NUMERIC
4.2.3	Mood swings	0 = mentioned, 1 = not mentioned
4.2.3.1	Mood swings begin (month)	NUMERIC
4.2.3.2	Mood swings end (month)	NUMERIC
4.2.4	Other	0 = mentioned, 1 = not mentioned
4.2.5	Improvements	0 = mentioned, 1 = not mentioned
