# Supplementary material for: YouTube and the implementation and discontinuation of the oral contraceptive pill: A mixed-method content analysis
Source: PLoS One. 2024 May 24;19(5):e0302316. doi: 10.1371/journal.pone.0302316 (PMC11125465; doi:10.1371/journal.pone.0302316)
Supplement: Attachment — Submitted filename: PONE-D-24-01385_reviewer.pdf [file pone.0302316.s005.pdf]

PLOS ONE

YouTube and the implementation and discontinuation the oral contraceptive pill: A mixed-method content analysis

--Manuscript Draft--

**Manuscript Number:**	PONE-D-24-01385
**Article Type:**	Research Article
**Full Title:**	YouTube and the implementation and discontinuation the oral contraceptive pill: A mixed-method content analysis
**Short Title:**	YouTube and the oral contraceptive pill
**Corresponding Author:**	Jana Niemann<br>Charite Universitatsmedizin Berlin<br>Halle (Saale), GERMANY
**Keywords:**	Content analysis; stopping; social media analysis; YouTube analysis, contraceptive pill, oral contraception
**Abstract:**	Background<br>Women living in high quality health care systems are more likely to use oral contraceptives at some point in their lives. Research findings have sparked controversial discussions about contraception in the scientific community and the media, potentially leading to higher rates of method discontinuation. Understanding the underlying motives for method discontinuation is crucial for reproductive health equity and future programming interventions. To address this question, this study aims to explore women's experiences of oral contraceptive use and discontinuation on YouTube.<br>Methods<br>A concurrent explanatory mixed-methods design was used to conduct content analysis of German YouTube videos. The information from 175 videos of 158 individuals was extracted through quantitative descriptive content analysis. 21 individuals were included in the qualitative content analysis.<br>Findings<br>The body was a recurring theme in the pill biographies. For example, women described bodily sensations as reasons for taking and stopping the pill. They also described positive and negative side effects while taking the pill and after stopping. The most common side effects of taking the pill mentioned by YouTubers were mood swings (76/158), weight gain (45/158), headaches (33/158), and depressed mood (45/158). The most reported symptoms after discontinuation were facial skin impurities (108/158), decreased mood swings (47/158), hair loss (42/158), and weight loss (36/158). Overall, women overwhelmingly rated their discontinuation experience as positive (87/91).
**Order of Authors:**	Jana Niemann, M.Sc.
	Lea Wichersiki
	Lisa Glaum
	Liane Schenk, Prof. Dr.
	Gertraud Stadler
	Matthias Richter
**Additional Information:**	
**Question**	**Response**
**Financial Disclosure**<br><br>Enter a financial disclosure statement that describes the sources of funding for the work included in this submission. Review	The author(s) received no specific funding for this work.

Powered by Editorial Manager® and ProduXion Manager® from Aries Systems Corporation

the submission guidelines for detailed requirements. View published research articles from *PLOS ONE* for specific examples.<br><br>This statement is required for submission and **will appear in the published article** if the submission is accepted. Please make sure it is accurate.<br><br>**Funded studies**<br>Enter a statement with the following details:<br>• Initials of the authors who received each award<br>• Grant numbers awarded to each author<br>• The full name of each funder<br>• URL of each funder website<br>• Did the sponsors or funders play any role in the study design, data collection and analysis, decision to publish, or preparation of the manuscript?<br><br>Did you receive funding for this work?	
**Competing Interests**<br><br>Use the instructions below to enter a competing interest statement for this submission. On behalf of all authors, disclose any competing interests that could be perceived to bias this work—acknowledging all financial support and any other relevant financial or non-financial competing interests.<br><br>This statement is required for submission and **will appear in the published article** if the submission is accepted. Please make sure it is accurate and that any funding sources listed in your Funding Information later in the submission form are also declared in your Financial Disclosure statement.<br><br>View published research articles from *PLOS ONE* for specific examples.	The authors have declared that no competing interests exist.

Powered by Editorial Manager® and ProduXion Manager® from Aries Systems Corporation

**NO authors have competing interests**<br><br>Enter: *The authors have declared that no competing interests exist.*<br><br>**Authors with competing interests**<br><br>Enter competing interest details beginning with this statement:<br><br>*I have read the journal's policy and the authors of this manuscript have the following competing interests: [insert competing interests here]*<br><br>* typeset	
**Ethics Statement**<br><br>Enter an ethics statement for this submission. This statement is required if the study involved:<br><br>• Human participants<br>• Human specimens or tissue<br>• Vertebrate animals or cephalopods<br>• Vertebrate embryos or tissues<br>• Field research<br><br>Write "N/A" if the submission does not require an ethics statement.<br><br>General guidance is provided below. Consult the submission guidelines for detailed instructions. **Make sure that all information entered here is included in the Methods section of the manuscript.**	N/A

Powered by Editorial Manager® and ProduXion Manager® from Aries Systems Corporation

**Format for specific study types**<br><br>**Human Subject Research (involving human participants and/or tissue)**<br>• Give the name of the institutional review board or ethics committee that approved the study<br>• Include the approval number and/or a statement indicating approval of this research<br>• Indicate the form of consent obtained (written/oral) or the reason that consent was not obtained (e.g. the data were analyzed anonymously)<br><br>**Animal Research (involving vertebrate animals, embryos or tissues)**<br>• Provide the name of the Institutional Animal Care and Use Committee (IACUC) or other relevant ethics board that reviewed the study protocol, and indicate whether they approved this research or granted a formal waiver of ethical approval<br>• Include an approval number if one was obtained<br>• If the study involved *non-human primates*, add *additional details* about animal welfare and steps taken to ameliorate suffering<br>• If anesthesia, euthanasia, or any kind of animal sacrifice is part of the study, include briefly which substances and/or methods were applied<br><br>**Field Research**<br><br>Include the following details if this study involves the collection of plant, animal, or other materials from a natural setting:<br>• Field permit number<br>• Name of the institution or relevant body that granted permission	
**Data Availability**<br><br>Authors are required to make all data underlying the findings described fully available, without restriction, and from the time of publication. PLOS allows rare exceptions to address legal and ethical concerns. See the PLOS Data Policy and FAQ for detailed information.	No - some restrictions will apply

Powered by Editorial Manager® and ProduXion Manager® from Aries Systems Corporation

A Data Availability Statement describing where the data can be found is required at submission. Your answers to this question constitute the Data Availability Statement and **will be published in the article**, if accepted.<br><br>**Important:** Stating 'data available on request from the author' is not sufficient. If your data are only available upon request, select 'No' for the first question and explain your exceptional situation in the text box.<br><br>Do the authors confirm that all data underlying the findings described in their manuscript are fully available without restriction?	
**Describe where the data may be found in full sentences. If you are copying our sample text, replace any instances of XXX with the appropriate details.**<br><br>• If the data are **held or will be held in a public repository**, include URLs, accession numbers or DOIs. If this information will only be available after acceptance, indicate this by ticking the box below. For example: *All XXX files are available from the XXX database (accession number(s) XXX, XXX.).*<br>• If the data are all contained **within the manuscript and/or Supporting Information files**, enter the following: *All relevant data are within the manuscript and its Supporting Information files.*<br>• If neither of these applies but you are able to provide **details of access elsewhere**, with or without limitations, please do so. For example:<br><br>*Data cannot be shared publicly because of [XXX]. Data are available from the XXX Institutional Data Access / Ethics Committee (contact via XXX) for researchers who meet the criteria for access to confidential data.*<br><br>*The data underlying the results presented in the study are available from (include the name of the third party*	All relevant data are within the manuscript and its Supporting Information files.

and contact information or URL).<br>• This text is appropriate if the data are owned by a third party and authors do not have permission to share the data.<br><br>* typeset	
Additional data availability information:	

Manuscript Click here to access/download;Manuscript;Manuscript.docx

Title

YouTube and the implementation and discontinuation the oral contraceptive pill: A mixed-method content analysis

Jana Niemann1,2, Lea Wicherski3, Lisa Glaum4, Liane Schenk1, Getraud Stadler5 and Matthias Richter6

Affiliations

1 Charité - Universitätsmedizin Berlin, Institute for Medical Sociology and Rehabilitation Science, Berlin, Germany

2 Martin Luther University Halle Wittenberg, Medical Faculty, Interdisciplinary Centre for Health Sciences, Institute of Medical Sociology, Institute of Medical Sociology (IMS), Halle (Saale), Germany

3 Osnabrück University, School of Human Sciences, Osnabrück, Germany

4 Bielefeld University, School of Public Health, Bielefeld, Germany

5 Charité Universitätsmedizin Berlin, Institute for Gender Research in Medicine (GiM), Berlin, Germany

6 Technical University of Munich, Department of Sport and Health Sciences, Munich, Germany

Correspondence

Correspondence to jana.niemann@charite.de

Abstract

Background

Women living in high quality health care systems are more likely to use oral contraceptives at some point in their lives. Research findings have sparked controversial discussions about contraception in the scientific community and the media, potentially leading to higher rates of method discontinuation. Understanding the underlying motives for method discontinuation is crucial for reproductive health equity and future programming interventions. To address this question, this study aims to explore women's experiences of oral contraceptive use and discontinuation on YouTube.

Methods

A concurrent explanatory mixed-methods design was used to conduct content analysis of German YouTube videos. The information from 175 videos of 158 individuals was extracted through quantitative descriptive content analysis. 21 individuals were included in the qualitative content analysis.

Findings

The body was a recurring theme in the pill biographies. For example, women described bodily sensations as reasons for taking and stopping the pill. They also described positive and negative side effects while taking the pill and after stopping. The most common side effects of taking the pill mentioned by YouTubers were mood swings (76/158), weight gain (45/158), headaches (33/158), and depressed mood (45/158). The most reported symptoms after discontinuation were facial skin impurities (108/158), decreased mood swings (47/158), hair loss (42/158), and weight loss (36/158). Overall, women overwhelmingly rated their discontinuation experience as positive (87/91).

Conclusions

The study identified key symptoms of oral contraceptive initiation and discontinuation by portraying the experiences of female YouTubers, adding valuable insights to the understanding of method initiation and discontinuation. Further research is needed to explore women's personal experiences with method discontinuation beyond the YouTube platform.

Keywords

Content analysis, discontinuing oral contraceptives, social media analysis, YouTube analysis, contraceptive pill

Introduction

The oral contraceptive pill (OCP) is one of the most important contraceptives in high-quality health care systems in Northern and Western Europe [1–3]. Since its introduction 60 years ago, women[1] have continuously complained about the side effects of the pill [4]. These have been supported by research, especially in recent years (e.g.[5–10]). However, most of these studies have reported associations with side effects such as depression, or venous thromboembolism (VTE) risk, not causality. This has led to increasing criticism, especially by (mainstream) media[2,11–13].

THE PILL AND (MAINSTREAM) MEDIA – A PROBLEMTATIC RELATIONSHIP?

History of the relationship of the pill and (mainstream) media

The dynamic between mainstream media and the OCP has a long history: for more than half a century, women's right to sexual self-determination and the pill's side effects have been discussed in the media[14,15]. An important example is the "Pill-Scare" of 1995[16–18]. Here, the dissemination and interpretation of scientific research regarding the risk of VTE associated with the Pill, lead to an increased number of pill discontinuations. This was followed by the individual contraceptive use, and increased unintended pregnancies and abortions[14,15,18]. Media-critical discourse continued to emphasize the increased risk of VTE in the 2000s and early 2010s. However, ignoring studies that found no increased risk[15]. In Germany, this was also taken up in the pill report[11] in 2015 and further disseminated by the media[19–21].

In 2016 and 2017, the media focused on the increased risk of depression[22,23], based two Danish studies[9,24]. These studies were, however, criticized in the scientific context due to methodological weaknesses[23]. In this wave of pill- skepticism, women shared their personal experiences with the pill under the #MyPillStory on social networking sites (SNSs)[25,26]. In the SARS-CoV-19 crisis, the VTE risk associated with the OCP was used as a comparison to the VTE risk associated with the Vaxzervria vaccine (formerly COVID-19 Vaccine AstraZeneca). This is a difficult comparison because the VTE

[1] We recognize that people other than cisgender women have menstrual periods and use birth control to prevent pregnancy. In this article, we understand the word "women" to be an inclusive term.

associated with OCPs is deep vein thrombosis and pulmonary embolism. Whereas the VTE risk associated with the AstraZeneca vaccine is cerebral venous sinus thrombosis[13].

In summary, there has been an underrepresentation of studies that do not show correlations with side effects in the media journalistic discussion of the OCP, and there has often been a lack of distinction between absolute and relative risks[15,22]. The scientific analysis and evaluation of online contraceptive information has therefore become particularly important.

Researching SNSs and (hormonal) contraception

With the advent of the Internet, the relationship between media journalism and health care decision making has increased significantly[14,27]. Increased access to online resources and SNSs, has expanded the range of information available to women and increased their autonomy to choose contraception[13,14]. Hence, they have emerged as powerful tool for health communication.

With this increase, there is also a need in exploring the content, narratives, and quality of online information regarding (hormonal) contraception. Vieth et al. [28] found that among German women the internet was the most common source of contraceptive information. Studies, for instance, researched the experiences birth control on YouTube[29–32], TikTok[33] and Reddit[34]. An analysis of German SNSs contraception content on YouTube, Instagram, and TikTok found clear shortcomings in the quality and completeness of information[35]. Also, a study by Alves and collogues[36], found that most of the websites mentioning the VTE risk associated with OCPS, do not refer to information from accredited health agency sources. In terms of health communication science, so-called health laypeople (users of a contraceptive method) can disseminate information on the Internet[37]. Döring et al.[35] showed that most German contraceptive content on YouTube, Instagram, and TikTok was uploaded by health laypersons, sharing personal contraception stories.

Informed choice regarding contraceptive methods is an important component of the Sexual and Reproductive Justice Framework. However, the biased media attention and misinformation might lead to higher discontinuation rates among OCP users.

ORAL CONTRACEPTIVE PILL DISCONTINUATION

OCP discontinuation can be defined as the initiation of a contraceptive method and the subsequent discontinuation of the method for any reason while there is still a risk of pregnancy. In a German study, 64.5% (n=1480) of those who used oral contraceptives at some point discontinued the method [28]. Simmons et al.[38] examined the cessation of contraceptive methods as part of a person's *Contraceptive Journey*. They defined key factors contributing to this decision: physiological factors, values, experiences, circumstances, and relationships. Discontinuation of OCPs, deprives the body of an external source of artificial progesterone and estrogen, resulting in a change in hormone levels in the body[39,40]. Current medical research on OCP discontinuation is limited to fertility restoration[41,42], fecundability[40], and cycle characteristics[41,43], and reasons for discontinuation[28,38,44].

Contributing to the narrative of OCP discontinuation, Kissling[45] explored posts of individual experiences on blogs and websites though a postfeminists lens. Further, Döring[22] conducted a content analysis of German posts about the contraceptive pill on TikTok and YouTube. She was interested in who the authors of the posts were, what the messages were about the contraceptive pill, and what the audience's reactions were. She found that on YouTube, most of the posts came from health laypeople. She characterizes them as "pill-weary women" who give autobiographical accounts of taking and stopping the pill. Viewers' reactions to these posts are mostly positive. Adding to this, a recent study[32] on birth control content on YouTube found in 50 videos most women talked about their discontinuation experience with the pill: the main outcomes after discontinuation were worsened acne (22%), improved mood (18%), cycle irregularities (14%), and increased energy (14%).

However, the health consequences of discontinuing OCPs in general and on social media have not been adequately studied in the German context. And, as noted by Inoue et al.[44], there is a lack of research on women's own experiences after stopping OCPs.

CONTENT ANALYSIS AIMS AND RESEARCH QUESTIONS

Since 2005, YouTube has established itself as a way for individuals to share information and present themselves online. It is a live video sharing platform that is constantly changing[46,47]. Based on Döring's[22] and Pfender's and Devlin's[32] analysis, YouTube videos uploaded by health laypersons,

provide an opportunity to scientifically examine these personal experiences and determine the health consequences of initiating and discontinuing OCPS.

The overall aim of this content analysis was to describe and quantify the observed personal physiological and psychological changes after initiation and discontinuing of contraceptive pill treatment of German-speaking YouTubers. We addressed the following research questions:

RQ1: For how many years did women stay on the OCP?

RQ2: What reasons for initiation of the OCP do women mention?

RQ3: What side effects of OCP implementation do women mention?

RQ4: How do women describe and evaluate their experiences during OCP implantation?

RQ5: What reasons for discontinuation of the OCP do women mention?

RQ6: What changes after OCP discontinuation do YouTubers mention?

RQ7: How do women describe evaluate their experiences with the discontinuation of the OCP?

Methods

DESIGN

The study evaluated video content data from the YouTube platform and was pre-registered at https://osf.io/mq3hj. It was designed as an (almost) simultaneous exploratory mixed-methods content analysis[48]. The qualitative and quantitative research strands were analyzed separately. The quantitative component was primarily used to present overall video characteristics and estimates of video content data. The qualitative component explored YouTubers' perceptions and beliefs as an in-depth analysis to complement the quantitative research.

SAMPLE

YouTube was initially searched from July 20 to 23, 2021. An updated search was performed on 2023/05/05. The internet browser and cookies history were cleared before the search. The sample was examined by using common searches for videos of German-speaking women who explained their personal experiences with the discontinuation of the pill. YouTube was searched using the autofill feature in the search bar (which uses an algorithm influenced by users' popular searches to automatically fill

search queries with root words)[49]. The following translated search terms were used: "stop taking the pill", "stop taking the pill experience", "discontinuation of the pill", and "discontinuation of the pill experience" (the exact German terms are provided in Supplementary Information (S1)). This search was performed following previous YouTube analyses (e.g.[46,49,50]). The videos were sorted by the relevance filter. For each search term, all videos were included. The videos were then checked for duplicates.

Only videos that met our inclusion criteria, as shown in Table 1, were included.

Table 1. Eligibility criteria

Inclusion criteria	Exclusion criteria
1) YouTube videos with a major focus on the discontinuation of the pill and its consequences.<br>2) YouTubers talk about their personal experiences with stopping of the pill.<br>3) The author herself must be the person talking about her experiences in the videos.<br>4) Video-data-content<br>5) Videos with a minimum length of 5 minutes.<br>6) German YouTube videos.	1) YouTube videos, where there is no major on the discontinuation of the pill (video formats such as Vlogs).<br>2) YouTubers that report on non-personal content that is (not) related to stopping of the pill.<br>3) A third party talking about the discontinuation of the pill.<br>4) Other media formats, such as podcasts.<br>5) Videos shorter than 5 minutes.<br>6) Non-German YouTube videos.<br>7) SHORTS, a short video format on YouTube.

If a If the YouTuber decided to upload more than one video about stopping the pill, two to three videos were included: The first uploaded video (most likely to contain relevant information about reasons for discontinuation or duration of pill use) and the second and/or last uploaded video (most likely to be the most recent version regarding immediate health consequences). If a YouTube video was uploaded with two women talking about their experiences, both individuals were included in the study. Different personal identifiers (see ethics) were assigned.

Figure 1 shows the flowchart of the search. A total of 1344 videos were initially collected by JN. After removing all duplicates, 591 videos were reviewed by JN and LW. After removing an additional 416 videos, a final number of 175 videos from 158 YouTubers were included in the sample for quantitative content analysis. All individuals could be identified as female.

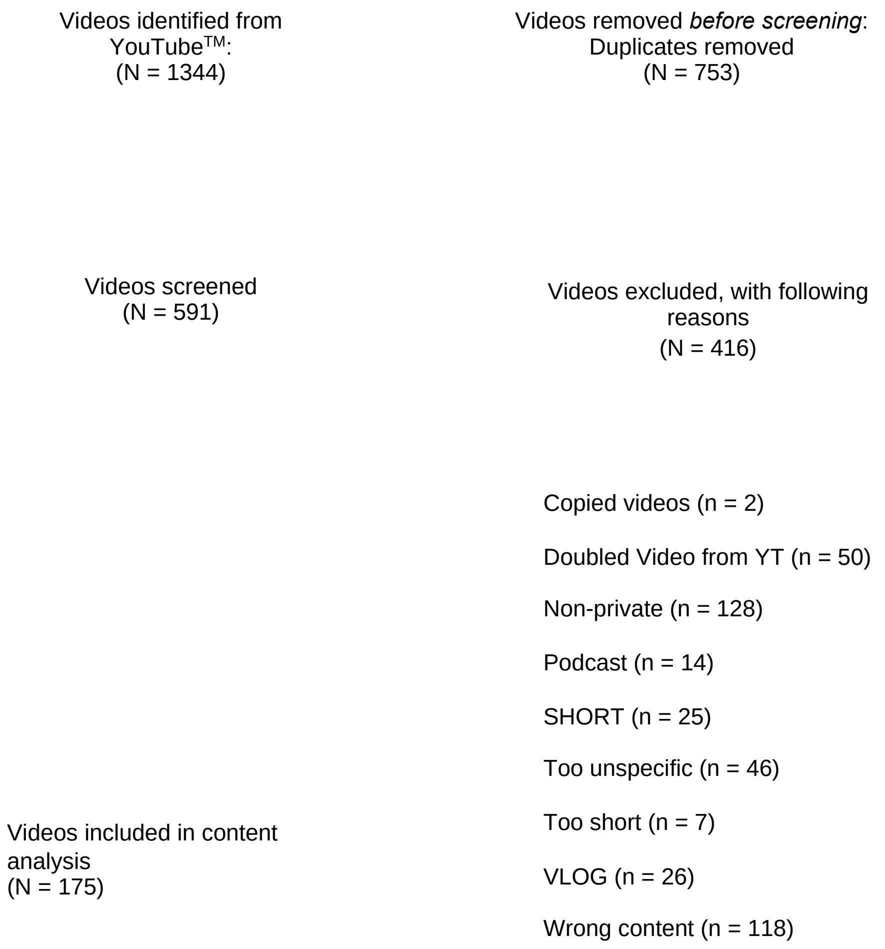

Fig. 1 Flow diagram of video selection process

Hereinafter, 18 videos were included as an initial sample for the qualitative content analysis. These were selected by JN and LW after reviewing all 175 videos, based on stratification factors, to represent the variety of experiences of the study sample:

- reasons for initiation and discontinuation,
- length of initiation,
- age at initiation and discontinuation,
- experienced side effects during intake and after discontinuation.

After JN and LW coded the initial sample, three additional videos were included to reach point of data saturation[51]. Resulting in a total qualitative sample of 21 videos.

PROCEDURE AND ANALYSIS OF THE CONTENT ANALYSIS

The unit of analysis for the media content was the uploaded videos (audio and text presentation) and the person(s) who reported on their personal experiences with stopping the pill on the online platform YouTube.

Procedure and analysis of the quantitative content analysis. A predefined, theoretically driven codebook (Supplementary Material XY) was developed. Quantitative content analysis consisted of coding content data related to the video (e.g., V001) and the person(s) reporting their experience (e.g., P001):

1. Typical YouTube **video characteristics**, including posting date, video length, number of views, likes, and comments.
2. **General content data** regarding the pill persistence and discontinuation, such as time of video recording after discontinuing the pill (weeks), length of pill persistence (years), age at initiation and pill discontinuation, second discontinuation, reasons for initiation and discontinuation (0 = not mentioned, 1 = mentioned), and current contraceptive method (0 = not mentioned, 1 = mentioned).
3. **Content data regarding the pill implementation**, including the mention (0 = not mentioned, 1 = mentioned) of improvements (e.g., facial skin) and side effects (e.g., migraine).
4. **Content data regarding the pill discontinuation**, including the mention (0 = not mentioned, 1 = mentioned) of the physiological and psychological changes (e.g., mood swings).

The translated codebook is available as supplementary information (S2). JN and LG independently assessed the intercoder-reliability of the codebooks based on 17 randomly selected videos from the sample. The Krippen-Dorff's Alpha reliability coefficient was calculated for all content variables in the codebook. The following average reliability value was obtained for the codebook: 0.813, indicating good measurement accuracy[52].

Data was collected by downloading the videos and the text presentation. These were transferred directly into MAXQDA 2020, where the material was coded by JN. The data were then transferred to an Excel spreadsheet for registration. Quantification was performed by JN using descriptive data analysis (frequencies and percentages) in STATA 14.2.

Procedure and analysis of the qualitative content analysis. This analysis consisted of coding the content data related to the person sharing their experiences (e.g., P001) in the uploaded video. After selection, the videos of YouTubers were transcribed verbatim. Their content was then analyzed by JN and LW according to Mayring's[53] qualitative content analysis. A category system was developed beforehand, according to which the content-related aspects of the material were examined. The category system is available as supporting information (S3).

The categories were developed based on the current state of research and by considering the codebook of the quantitative research strand. Further subcategories were created based on the research questions and inductively from the material. Subsequently, coding rules were defined, and anchor examples were added. The videos were transcribed and coded in MAXQDA 2020.

ETHICS

We included only adult YouTubers who are aware of their publicity (e.g., through follower numbers) and the publicly available works. The German Society for Online Research[54] states that the use of publicly available data for scientific research is ethically appropriate even without informed consent.

To minimize the risk of identification, all videos and individuals were pseudonymized at the beginning of the data collection. This makes it impossible or very difficult to identify them in the research process: All videos were assigned a video identification number (e.g., V001). All persons have a personal identification number (e.g., P001). The personal data (e.g., title, YouTube channel name) were separated from the factual data (e.g., time of posting, views) and stored on different media.

Results

The 175 Videos analyzed in this study were posted between 2014 and 2023. The mean video length was 14.8 minutes. The detailed general characteristics are available in Supplementatry Table 1.

GENERAL CHARACTERISTICS OF THE CONTRACEPTIVE PILL PERSISTENCE

The mean duration of OCP use was 8.2 years. Women started using oral contraceptives at a mean age of 14.8 years. The mean age of discontinuation was 23.1 years. See Table 2 for details.

Table 2 General characteristics of pill persistence

Characteristics	Min	Max	Median	Mean	Standard deviation
**Time since discontinuation (weeks)** (N = 120)	0.0	108.0	12.0	15.9	19.4
**Persistence**					
Length (years) (N = 119)	1.0	27.0	7.0	8.2	4.3
Age initiation (N = 119)	12.0	23.0	15.0	14.8	1.4
Age discontinuation (N = 95)	17.00	37.00	22.7	23.1	3.5

THE OCP IMPLEMENTATION AND DISCONTINUATION

The following results show that embodiment and effects on the body play a central role in pill use and discontinuation. This recurs in the detailed descriptions of the psychological and physical aspects of each phase of uploaded the pill autobiographies.

Characteristics of the pill implementation

Table 3 details all information of the pill implementation. 57 of 158 YouTubers changed their type of pill during the implementation period. Aspects of this are that switching may have occurred because of side effects of the pill (P018, P149), side effects occurred after switching (P010, P100), or side effects persisted and worsened after switching (P149).

The reasons for taking the pill were varied and included facial skin (35/158), contraception (30/158) and/or painful menstruation (27/158). Many felt that the pill was the only option available to them and were unaware of alternative contraceptive methods. The women expressed the normality of the pill as a solution for treating facial skin impurities (P002) and the recommendation of the gynecologist for menstrual cramps (P010). According to the average age at initiation, it was stated that pregnancy was not an option due to the young age and that the pill was considered a safe method of contraception (P006, P018, P149).

Positive side effects during implementation

Overall, 45 of the 158 women experienced improvements while using OCPs. The improvements were consistent with the main reasons for initiation: facial skin (25/158), painful bleeding (19/158), and decreased bleeding (12/158). The contraceptive pill appears to be particularly convenient as it combines the treatment of medical problems such as painful bleeding with the need for contraception (P007, P010).

Negative side effects during implementation

Physiological side effects were reported by 101 of all YouTubers. The most common side effects were weight gain (45/158), headache (33/158), and water retention (30/158). Headaches seemed to be very distressing and interfered with women's lives (e.g., P001, P007, P100). 103 of all women reported psychological side effects. The most common psychological side effects were mood swings (76/158), depressed mood (45/158), and deterioration of libido (31/158). Mood swings were characterized as being moody, irritable, unrelaxed, aggressive, or angry (e.g., P054, P032, P010). Women also reported feeling like something was missing or feeling emotionally numb while on the pill.

Table 3 Pill implementation

Generation	Mention % (n/N)	Example (Qualitative)
**Switch between pills**	36.1 (57/158)	“He prescribed me another pill and from then on I only had problems with the pill. All the side effects that I had before were much worse and I only really noticed them once.” *(P010)*
**Reasons for initiation***		
Facial skin	22.2 (35/158)	"[...] I really took it at that time because I had bad facial skin and at that time it was just like, if you have bad facial skin, just take the pill. And nobody thought too much about it, it was just normal and of course parents were like, okay, then take the pill before you get pregnant or something. And my facial skin actually got better and I was happy [...]" *(P002).*
Contraception	19.0 (30/158)	“After my first menstruation I started taking the pill, and at that time also for the purpose of contraception. Before that, I had already tried using a condom, but to be honest, I didn't really feel safe with it myself.” *(P149)*
Painful menstruation	17.1 (27/158)	"And for me, it wasn't about contraception at all, but I had very bad stomach pains every time I had my period, so I missed about three days a month. And like so many others, my gynecologist recommended the pill.” *(P010)*
Gynecologist	5.1 (8/158)	/
Other	17.1 (27/158)	“A lot of people may know that I had an abortion when I was 16 and I didn't say okay, I never want to have to go through what I went through there again. That's why I then started taking the pill” *(P148)*
**Improvements***	28.5 (45/158)	/
Facial skin	15.8 (25/158)	“[...] I got better facial skin quickly, although I have to say that when I went to the gynecologist, I also said that I would like to get a pill that would really improve my facial skin and my facial skin. So, I mean, I got a pill against acne and therefore my facial skin quickly improved accordingly and that was of course totally great.” (P100)
Painful bleeding	12.0 (19/158)	“I noticed right after the first month that my period was a lot less and it hurt a lot less and I was like oh my God (.) thank you that they invented something like this because it was really bad for me I had really bad pain". *(P001)*
Decreased bleeding	7.6 (12/158)	“Of course, the pill also had some positive effects. I had less heavy periods.” *(P010)*
Other	3.2 (5/158)	/
**Physiological problems***	63.9 (101/158)	/

Weight gain	28.5 (45/158)	"I had gained weight once because of it. Like I said, it didn't bother me that much. But that was already about eight kilos that I had gained. Just steady, steady, so there was no stopping it." *(P018)*
Water retention	19.0 (30/158)	"So a week before my period, I had so much water retention [...]" *(P079)*
Weight gain breast	8.9 (14/158)	/
Headache	20.9 (33/158)	"But at some point when I was 17, I noticed that I had extremely bad headaches all the time. Really, it was so bad for me. I used to just think I stayed home for two days because I had such stomach pains and at that point at 17 I stayed home because I had such bad headaches that I thought I had a migraine, what's wrong with me?" *(P007)*
Migraine	9.5 (15/158)	"So, in general, I have to say that with the pill, I had constant headaches, I really suffered from migraine attacks." *(P140)*
Exhaustion	9.5 (15/158)	"[...] when I was on the pill, I was super tired, super floppy, and really fell asleep at 9:30 in the morning because I was just so exhausted." *(P048)*
Spotting	5.1 (8/158)	/
Pain legs	5.1 (8/158)	"I have always had severe cramps in both legs. When I sat for a long time, it was like an endless cramp in my leg. It felt like dogs were biting me. So yes, but of course I thought it was normal." *(P140)*
Other	43.7 (69/158)	"I felt sick a lot. I had this nausea a lot, and I don't know, it was all kind of weird, and I thought about it a little bit and I thought, okay, what could it be?" *(P007)*
**Psychological problems***	65.2 (103/158)	/
Deterioration of libido	19.6 (31/158)	"Loss of libido, for example, is so extreme with me that I actually have no desire. So, I take this contraceptive and I have no desire for sex. So, you think, okay, what am I taking it for? Because if it doesn't do what it's supposed to do, I don't know, it's questionable." (*P119)*
Depressive moodiness	28.5 (45/158)	"And that was what was missing. I often felt in very, very positive situations, when you should feel very, very happy, that something was missing. So, I knew rationally, okay, I should be very, very happy now, I should be totally happy now, but inside I just didn't feel it and it was totally weird. But then a lot of times I just blamed it on the fact that I'm just getting older and maybe you just get a little bit of a number when you get older." (*P149)*<br>"I don't know if the depression I got when I was 19 was triggered by the pill. But there is a good chance that I am one of the 23% of women who suffer from depression triggered by the pill." (*P010*)
Mood swings	48.1 (76/158)	"I was super quick to get irritated; I was super quick to get bitchy when I didn't like something. So, I'm not really like that and I didn't want to be. But somehow, I did. The pill just made me super irritable and super moody before my period." (*P079)*
Other	11.4 (18/158)	"But then when I was 18, 19, I think, it started so slowly that I developed anxiety and I started having panic attacks and my body felt kind of weird." *(P044)*

*Double nomination possible

Characteristics of the contraceptive pill treatment discontinuation

As described in Table 4, the main reasons given by YouTubers for discontinuing oral contraceptive use were experienced side effects (74/158), increased awareness of the topic (21/158), and the desire to stop taking hormones (34/158). Qualitatively, side effects that lead to discontinuation could include a tipping point where a user can no longer tolerate the side effects. In particular, the severe impact of headaches while taking the pill was reported as a side effect leading to discontinuation (P001, P007, P018). 27 of all YouTubers reported discontinuing the pill for the second time. Reasons for resuming the pill were changes (e.g., facial blemishes) after the first discontinuation. However, as one YouTuber explained in P018, the side effects of the pill were even worse, which then led to the final discontinuation of the method. Ultimately, former users of the pill had a variety of reasons for stopping, but all prioritized their overall health and well-being.

Improvements after discontinuation

Of 158 YouTubers mentioned 54 physiological and 82 psychological improvements discontinuation. The most common improvements mentioned were weight loss (37/158), disappearance of headaches (23/158), decreased fatigue (13/158), decreased mood swings (47/158), increased libido (40/158), and decreased depressive mood (19/158). Women reported that discontinuation improved their psychological mood: They felt more relaxed and had a more positive attitude (P001, P002, P036, P048, P054). They also reported a return or increase in their libido (P002, P100, P140, P149). It was also expressed that many did not expect how extreme this return is (P010). They report feeling more in tune with their bodies and experiencing a greater sense of euphoria after weaning.

Negative changes after discontinuation

In general, 123 of all women experienced negative physiological and 32 psychological changes after discontinuation. An increase in facial skin blemishes the most reported negative physiological change after discontinuation (108/158). This was followed by hair loss (42/158), painful menstruation (36/158), and menstrual cycle irregularities (31/158). Negative psychological changes included mood swings (16/!58) or the onset of premenstrual syndrome (PMS) (10/158). The YouTubers claimed that their facial skin was never as bad as it was after stopping the pill. They mostly described their facial blemishes as "normal" and not as severe as in people with "real" acne. Conversely, they also described the onset

of facial skin deterioration as a major challenge and burden. Due to the strong identification with the facial skin and the external appearance, this becomes a problem. Thus, good make-up and the thought that others also have facial skin imperfections should help to conceal and accept one's own facial skin, which is seen as a flaw (P002, P018, P100). A minority of women stated that discontinuing the product caused a worsening of their psychological state (P085).

Evaluation of contraceptive discontinuation

Overall, 87 of 91 YouTubers rated their experience of discontinuation as positive. The women reflected on the strong influence of the pill on their bodies and health (P002, P010). They describe how happy they are to have freed their bodies from artificial hormones (P079). And that they are more in tune with their bodies and more aware of them (P010, P044, P100, P149). Words that appear frequently in the conclusions are "liberated" and "freer". For example, women feel freer after weaning, even though they say it is difficult to put into words (P032, P036, P054).

Current contraceptive method

The most common contraceptive currently used by YouTubers is the condom (26/84). This is followed by the copper IUD, chain, or ball (24/84) and the natural family planning (NFP) method (14/84).

Table 4 Pill discontinuation*

Generation	Mention % (n/N)	Example (qualitative)
**Reasons for discontinuation***		
Experienced side effects	46.8 (74/158)	"For one thing, I was really super tired and totally exhausted a lot of the time, even though I was eating healthy. [...] I had these headaches, and I don't know, for me it was just like there were so many things and so many issues. I always had this tight feeling in my chest and there were so many issues and thoughts in my head that I just really said, you know what, from one day to the next I'm going to take the pill all the way through this month, then I'm going to wait until I get my period and then I'm not going to take the pill anymore.." *(P007)*
Increased awareness	13.3 (21/158)	"The reason why I stopped was because I saw it with so many other people. So it was just kind of such a hype in the blogger world and in the YouTuber world that you stop taking the pill because yeah, because they didn't want to take hormones anymore. And then I heard that a lot of people were super happy with it and that a lot of people felt liberated and just had a much better mood and then I thought to myself, this is exactly what I want." *(P036)*
Fear of side effects & long-term consequences	8.9 (14/158)	"Let's put it this way, and especially if I were in my own facial skin, if I were affected by it myself, I wouldn't want it. I don't want thrombosis; I don't want hypothyroidism." *(P083)*
Exchange with friend	7.0 (11/158)	"I didn't come up with it myself, but my boyfriend at the time sent me an article and said, "Hey, somehow it turns out that a lot of pills increase the risk of thrombosis quite a bit. Check if your pill is one of them." *(P010)*

Desire to stop taking hormones	21.5 (34/158)	"[…] when I was no longer in a relationship, I decided I just wanted to stop taking the pill and see how my body felt without the hormones. *(P149)*
Own health	5.0 (8/158)	"I did it just for me, so just for me and my health and my body and so on." (P006)
Other	26.6 (42/158)	"It's not a great feeling. You don't really feel like a woman. It is. It's you. That's a really weird way to put it. I don't think you can say that you don't feel like a woman, for God's sake. But something is missing. There's something missing. And you can't put your finger on it because you don't know exactly what it is anymore." *(P083)*
**Second discontinuation**	17.1 (27/158)	"At some point, however, I started having problems that were very noticeable. I'll get into that in a minute, and then I just stopped taking it. A little later I started with another pill, which was also much weaker dosed. But I had severe problems again, extreme headaches, and then I stopped taking it. And that was it for me and this method of contraception. I never took the pill again." *(P018)*
**Improvements***		
Physiological	34.2 (54/158	/
Weight loss	23.4 (37/158)	"And I could definitely see how I lost those eight pounds on my stomach, thighs, and face in those eight weeks. ". *(P018)*
Decreased fatigue	8.2 (13/158)	"Since I stopped taking the pill. It's only been about half a year that I'm really up super late, I'm doing really well in the evenings, and I don't feel as drained as I used to." *(P048)*
Disappearance of headache	14.6 (23/158)	"And then what I also noticed enormously after I stopped taking the pill was that my headaches were gone. [...] I always had headaches and I always took pills because I couldn't stand it. When I stopped taking the pill, I didn't have headaches anymore. I don't have headaches anymore [...]" *(P002)*
Disappearance of migraine	6.3 (10/158)	"Because when I stopped, it was all gone. I never had migraines again, and I had them really really bad, so I just couldn't leave the house all day on the first day of my period, I had to lie in bed in my dark room." *(P036)*
Other	6.3 (10/158)	"So, first of all, it was definitely relatively quick after I stopped the pill that any side effects I had were gone. So the eye twitching was gone." *(P018)*
Psychological	51.9 (82/158)	
Increased Libido	25.3 (40/158)	"About a month or two after stopping the pill, my libido finally came back [...] I didn't expect it to be so extreme. It was like one day to the next. So from no desire at all to such a strong desire ((raises arm above head)) and I didn't even know I could have such a libido. Without going into too much detail now, but I had an almost constant desire for sex." *(P010)*
Decreased mood swings	29.7 (47/158)	"I also had very, very blatant mood swings in the early days. [...]. In the meantime it has really gone away and I also notice (..) everything is cool. And I'm really relaxed (..) so it's no longer this, this 180 feeling in me, it's so really so okay". *(P001)*
Decreased depressive moodiness	12.0 (19/158)	"From one day to the next, I was no longer sad, or simply no longer in a depressed mood. [...]. I was happy again. I could feel joy again." *(P036)*
Other	2.5 (4/158)	/
**Problems**		
Physiological	77.9 (123/158	
Hair loss	26.6 (42/158)	"I had real clumps of hair in my hand after washing my hair, and there was hair everywhere. Until I had real bald spots on my head [...]" *(P036).*
Greasy hair	15.2 (24/158)	"[...] my hair got greasy faster." *(P010)*
Facial skin impurities	68.4 (108/158)	"Sometimes I have to fight so hard with my facial skin. So even when I wear makeup now, you don't see it so much. But some days, for example, I only have three pimples and I go to sleep and wake up the next day and suddenly I have new pimples. It's just not a joke, it's so blatant sometimes. [...] I think it's a bit of a shame that I identify so

		much with my facial skin. So I always try to really let it go, because I think to myself: "This is just a facial skin and no idea, my facial skin is not supposed to be perfect." So, I mean, also, it's such a difficult subject, I think. Because on the one hand you see people with perfect facial skin all the time and you and maybe they use contraceptives. But I always try to isolate myself a little bit from that [...]". *(P100)*
Oily facial skin	8.9 (14/158)	"I got oily facial skin, I got really shiny. I didn't get any pimples, thank God". *(P001)*
Hormonal acne	5.7 (9/158)	/
First period		
1st month	22.2 (35/158)	/
2nd-5th month	6.3 (10/158)	/
6th-12th months	3.2 (5/158)	/
Cycle irregularities	19.6 (31/158)	“At first nothing happened. Really nothing. I didn't feel any different. I had no bleeding, nothing. I waited for two or three months and then at some point I went to my gynecologist and asked what was going on. My gynecologist was actually very calm and cool..” *(P010)*
Strong menstruation	13.9 (22/158)	" Of course, the period is heavier, but it is within a healthy range, I would say. So not that it would be extremely negative, but everything is still in the green zone, I would say.” *(P048)*
Painful menstruation	22.8 (36/158)	"[...] I had unbelievable pain again when I had my period" *(P085)*
Migraine	10.3 (15/158)	"The first week was really weird. I had such bad migraines, my head was completely full. I got up every day with a migraine." *(P140)*
Hair growth	5.7 (9/158)	"I've also noticed that my body hair has increased in places where I didn't really have any before. I've got such light beard hair, and also my hair on my legs, etc. has kind of grown faster. *(P010)*
Other	21.5 (34/158)	"I got cysts in my breasts, not one or two, but sometimes three in one breast, but they were not bad, just deposits in my body". *(P119)*
Psychological	20.3 (32/158)	/
Mood swings	10.1 (16/158)	"I felt that all those side effects that most people have from the pill, like mood swings and even mild depression, came to me the moment I stopped taking it. Well, not right away, of course, but after a few weeks or months when I stopped taking the pill. I didn't feel like doing anything. I just felt really bad. I felt such a complete lack of drive and I didn't want to do anything. I didn't want to see anybody.” *(P085)*
PMS	6.3 (10/158)	"Now that I'm off the pill, I feel but the last times I had my period always mood swings, but so negative stop somehow that I sometimes do not want the Stefan comes too close to me. Everything is often too much for me during my period. [And after my period I am the most relaxed and happy person again. So yeah, I find it pretty intense and I just don't know that about myself from before". *(P048)*
Other	12.7 (20/158)	/
**Conclusion discontinuation**		
Negative	3.3 (3/91)	"I finally went back on the pill after about four months because I was feeling so bad physically and psychologically that I figured I'll just try it and if it doesn't work then I'll have to change something else anyway. The bottom line is that now, two, three months later, I'm fine. So, I don't know, maybe it's just a placebo effect. Maybe it's something completely different or maybe my body just can't do without hormones anymore and has gotten so used to it that it somehow doesn't work without them. Which is not healthy either, I know. So this is definitely not a permanent solution. [...] But at least now it is my solution until I find something else. All my problems are definitely gone. I feel much better physically and mentally.” *(P085)*
Indifferent	1.1 (1/91)	/
Positive	95.6 (87/91)	"So the conclusion I can draw from all of this is [...] I don't regret it at all because my body is free of artificial hormones, I don't have headaches anymore, I'm calmer, I'm more relaxed, I'm much fitter, I feel better, I'm definitely healthier. I definitely know that it's much

		healthier for my body not to be pumped full of hormones every time [...]". *(P002)*
**Current contraceptive method**		
Condom	31.0 (26/84)	"[...] that's why I don't have any other contraceptive method like a condom now. I actually like it quite a lot. With a condom, you can always check to see if it leaked or not. And the morning-after pill would still be a hormonal option, but okay. I have to live with it now if I don't want to get pregnant." *(P083)*
Copper IUD/Chain/Ball	28.6 (24/84)	"The most important thing for me personally in a contraceptive method, besides the fact that it should be hormone-free, is safety, and I came across the T-coil and the copper IUD and decided to use them." (P018)
NFP	16.7 (14/84)	"[...] you can also use NFP for contraception. And because of the security that I had gained, I knew that the method really worked. Because I was honestly a little skeptical at first whether it would work so well with the body tracking, but then I was able to determine it, as I said, totally well, and that was honestly a pretty cool feeling. Yes, I talked to my partner about using it for contraception. I explained the method to him because it was very important to me that he knows what I'm doing and that he decides that it's okay for him that we use the knowledge for contraception. And since then we have been using NFP for safe, hormone-free contraception, which means that we have sex without a condom on the non-fertile days and with a condom on the fertile days." *(P149)*
Other natural contraceptive method	9.5 (8/84)	"We actually just do it by calendar. That means I look at when I ovulate. I look at when I could get pregnant, when it is most likely, and I abstain from sexual intercourse completely." *(P140)*
Other	14.3 (12/84)	"I am currently back on the pill. Just so that times so for info because I as I met my boyfriend back then [...]" *(P119)*

*Double nomination possible

Critical views on the contraceptive pill treatment.

Looking back on their time on the pill, women also expressed critical views. It was emphasized that the first sexual experiences in life were made under the influence of the pill (P010, P044). As one YouTuber explained:

> "One of the things I think is very fatal about very young girls being prescribed the pill is that many start taking it before they even have a sex life. That's exactly what happened to me. That is, every sexual experience I had was under the influence of the pill, so I didn't even know what sex and my sexuality would be like without the pill. So for years I thought a lot of things were normal. (P010)

The pill was also seen as a moneymaker for the medical/pharmaceutical market (P032) and as a beauty product (P119, P149). The method of prescribing hormones to young girls at the beginning of puberty was questioned. And they criticize the fact that the contraceptive pill was/is prescribed as a solution to non-contraceptive problems such as facial blemishes or cycle irregularities. As this woman explains:

> "And then I really wonder if it's necessary to try to influence that with drugs and hormones. Or maybe you could approach it differently or go in the direction of facial skin care and see a dermatologist to see if that might help [...]. Instead of just taking these hormones, especially at a young age, to get a better picture, I find it a little bit critical, to be honest, because I always think to myself, I don't know, it's coming from something else in your body. And you're just not treating the cause, you're just treating the problem at that moment." (P119)

The general cost of contraception as a female burden was also mentioned. In P130 the woman says that this is not an issue between man and woman. Instead, public health insurance should cover the costs of contraceptive methods in general.

Recommendations for discontinuing the pill.

In several videos, women described different aspects of stopping the pill. The first recommendation was that women should not stop taking the pill uninformed and indiscriminately (P018, P050). Women should make up their own minds, seek medical advice (P002, P007) and not stop taking the pill over-night, especially if it is being used for contraception. It is also mentioned that both starting and stopping the pill is a very individual matter (P018, P032).

The second recommendation emphasizes that discontinuation is an individual decision. The YouTubers explain that the choice of contraception is a private matter (P006). You should not be talked into it (P018, P054) and make your own informed decision:

> "It doesn't have to be for everyone and I would say to you don't let anybody talk you into something if you feel comfortable with it and it doesn't have to be the pill, it actually applies to all situations in life. If you feel comfortable with something and you think it works for you, then stick with it. Don't let anybody talk you into something that you don't feel is right for you." (P085)

Finally, some YouTubers also recommend stopping the pill (P140, P010). They were so positive about their experience of stopping, describing how it is better to do without:

"That's why I can advise every woman and every girl to think about whether you take the pill, whether you start taking it or whether you continue taking it. Do some research. Pay attention to your body, pay attention to possible side effects, and think twice. So if you are thinking about going off the pill, or if you are thinking about going off the pill, I wish you perseverance and I promise you it will have a positive impact on your life and you will not regret it. (P010)

Discussion

Quantitative analysis of YouTube videos showed that the duration of contraceptive pill use was approximately eight years (**RQ1**). The main reasons for initiating oral contraceptive treatment were to improve facial skin problems, as a contraceptive method, and for painful menstrual cramps (**RQ2**). The most common side effects during oral contraceptive treatment were mood swings, weight gain, headache, depressed mood, decreased libido, water retention, and migraine (**RQ3**). Although OCPs combined both the treatment of medical problems and the need for contraception, women described the adverse effects as burdensome (**RQ4**). Women discontinued OCPs mainly because of side effects, the desire to stop taking hormones, and increased awareness of the issue (**RQ5**). The most common health effects after discontinuation were as follows Facial skin impurities, hair, and weight loss, as well as decreased mood swings and increased libido (**RQ6**). Most women rated their discontinuation experience as positive. They reported feeling more connected to their bodies and freer after stopping (**RQ7**). In addition, this analysis showed that the bodily experience seems to be an important component in the pill autobiographies told in YouTube videos. To the authors' knowledge, this is one of the few studies of the personal views and experiences of women who have discontinued the pill. The results of this study are consistent with scientific research on the positive and negative side effects[55–57] of OCPS, and the reasons for discontinuation[28,44,58].

Puberty is the time when most girls in quality health care systems visit a gynecologist for the first time. This may be for specific health problems, such as menstrual cramps, acne and cycle irregularities, or for contraceptive advice[59,60]. In our sample, most women initiated OCPS during puberty, an age when physical and hormonal development from girl to woman is not yet complete. The contraceptive pill

therefore acts as a drug in an organism that is not yet fully developed[61]. In this context, it is important that health professionals provide information and advice on the costs and benefits of contraception.

In general, the side effects reported by the YouTubers during oral contraceptive pill treatment are consistent with the associated side effects reported in other studies[39,56,62] and listed on the OCPS package inserts[63]. However, the high number of side effects experienced by YouTubers was striking. This may be because YouTubers may be more likely to report discontinuing the pill if they experienced side effects while either taking or discontinuing the pill. Thus, there may be a publication bias in the YouTube videos and the results should be interpreted with caution.

The strata of this analysis are characterized by their young age at quitting. This may reflect the medium of YouTube as a YouTube consumer, which is specific to the sample of this study. However, it could also reflect the fact that women stop using the pill at a relatively young age. In particular, compared to the average age of women at first birth in the European Union (29.4 years) and Germany (31.2 years) in 2019[64].

The top two reported reasons for discontinuing oral contraceptive use were side effects and the desire to stop taking hormones. This is consistent with the quantitative literature, where side effects[28,44,58] or concerns about long-term effects[28] are predominantly cited as the main reason for discontinuation. Also, Pfender's and Devlin's[32] analysis, which reported that 22 out of 50 YouTubers discontinued hormonal contraception for "being more natural". A 2015 systematic review of medical and epidemiologic studies[44], which examined contraceptive discontinuation, found that pregnancy was among the top two reasons for discontinuation. This discrepancy could be explained by the young age at discontinuation of the sample included in this study. However, it may be the other reasons for discontinuation mentioned above that prompted the women to create a YouTube video.

Scientific studies show that women, in particular, are under social pressure when it comes to their external appearance[65–67]. For instance, even mild forms of acne are associated with a reduced quality of life[68]. The study showed that facial skin deterioration was the most common symptom after discontinuation of oral contraceptive therapy. Even the mild symptoms reported by women were associated with reduced self-esteem and discomfort. Accordingly, even medically harmless facial skin complaints after discontinuation represent a major psychological burden for female YouTubers. The literature also

describes that women, on average, experience longer cycles and more variability in cycle length after discontinuation [42,43,69]. This is consistent with the experience of female YouTubers. The contraceptive pill has also been questioned and studied for its possible negative association with mental [10,24,56,70] and sexual health[10,57,62]. Research on these topics should still be interpreted with caution[10]. Although it did not seem to be a central theme among the YouTubers, women in our sample reported mood swings and a decrease in libido as side effects of OCPs and their improvement after discontinuation. Interestingly, the prescription of OCPs before young girls have a regular sex life was also criticized. Despite the negative impact of health symptoms after discontinuation, such as facial skin deterioration, the women overwhelmingly experienced discontinuation as positive.

When OCPs are discontinued for reasons other than pregnancy, the use of effective contraceptive methods is very important for reproductive health. Otherwise, women are at high risk of becoming pregnant. The data show that most YouTubers switch to condoms or a copper implant (IUD, ball, or chain). However, NFP, a modern Fertility Awareness-Based (FAB) method, is also used quite frequently. Both copper implants[71] and modern FAB methods are effective ways to prevent pregnancy[72,73]. In addition, research shows that women have an increasing desire for more autonomy in their contraceptive choices[74–77]. Informed women's choices are associated with better contraceptive adherence and fewer contraceptive failures[78]. However, there may be differences in the counseling and recommendations of health care providers regarding contraceptive methods. For example, research by Irala et al.[79] suggests that OCPs and the IUD were predominantly chosen as a contraceptive method because of the doctor's suggestion/recommendation rather than because of their own desire. The opposite was true for the male condom or modern FAB methods. A German study found that participants said they would like to be better informed about the pill. And that women who received information from their gynecologist were more likely to feel well informed (OR 1.59, CI: 1.10-2.30) than those who received information from the Internet[28]. To increase autonomy in contraceptive choice and ensure contraceptive effectiveness, health care providers should be made more aware of alternative contraceptive methods other than OCPs.

Looking at all the results, the body and bodily perception seem to play a central role in all phases of the pill autobiography. The embodiment[79] is reflected in the reasons for initiating and weaning OCPs, in the experienced side effects, and in the changes that occur after stopping the pill. This role of bodies and contraception is consistent with other research: for example, the pill and other contraceptive methods have led to women having greater control over their reproductive bodies[80, 81]. And also, the importance of the (gendered) body in women's contraceptive decision-making[36, 80, 82].

FUTURE RESEARCH DIRECTIONS

The framing of health information can influence health-related beliefs, attitudes, and behaviors[80]. The increase in information and interpretation of scientific evidence in the media can improve informed contraceptive choices. Conversely, they can also negatively affect public health through misinterpretation of scientific evidence[27]. We emphasize the need to further investigate the relationship between SNSs and individual contraceptive method choice (discontinuation). There is a need for longitudinal and qualitative research to examine and explore the underlying processes in the future. In the context of our study design, it would be interesting to investigate if and how the private experiences of influencers on YouTube and other SNSs platforms (e.g., Instagram & TikTok) are used as health information regarding method discontinuation choices. In particular, because women who seek contraceptive information online appear to have lower levels of trust in information provided by their gynecologists[28]. It would also be interesting to explore the underlying motivations and phenomena of uploading such private information publicly. Especially in the context of the dynamics of self-presentation and online marketing.

STRENGTHS AND LIMITATIONS

The methodological strength of this study was its mixed methods design. It allowed for a greater contextual understanding of the descriptive data presented through the qualitative analysis. In addition, the findings represent the personal views and experiences of the women. These are naturally expressed and not biased by the research design itself. However, because these findings are based on YouTube, they may have limited generalizability and transferability to the general population. The sample may be biased because the women who post content on YouTube may be different from the average woman who discontinues hormonal contraception. This recruitment method also did not allow us to collect important

background information (e.g., socioeconomic status, gender, ethnicity). In addition, women who experience severe side effects during use and health symptoms after discontinuation may be more likely to post their experiences on YouTube than women with no side effects or symptoms. Thus, we don't know which of the data are factual and which might be influenced by self-presentation on social networking sites. The reach of the videos may also have led women to be less open about this sensitive topic than in face-to-face interviews, for example. This study is also limited to the content of what women report in their videos. For example, it cannot consider changes in life circumstances (e.g., change of life/sexual partner, facial skin care routine).

Conclusions

This content analysis of YouTube videos portrayed the personal implementation and discontinuation experiences of OCPS among female YouTubers. In doing so, the study provided valuable insights into the lived experiences, perceptions, and opinions of women who discontinued OCPS in the context of quality health care systems. Future qualitative and quantitative research is needed to provide information on the motivations, subsequent health symptoms, and health care needs associated with discontinuing OCPS.

Declarations

Availability of Data and Materials

The datasets used and analyzed during this study are available from the corresponding author upon reasonable request.

Abbreviations

IUD: Intrauterine devices

OCP: Oral contraceptive pill

SNSs: Social networking sites

VTE: Venous thromboembolism

Ethics approval and consent to participate

Not needed.

Consent for publication

Not needed.

Author contributions

JN conceptualized the study. J.N. and L.W. screened the YouTube-Videos. J.N .conducted the quantitative analysis. J.N .and L.G. coded the quantitative data for the intercoderreability. Both J.N. and L.W. conducted the qualitative analysis. JN wrote the initial draft for the manuscript. L.W., L.G., L.S., T.S., G.S., and M.R. provided substantial feedback on the idea of the content analysis and on different versions of the manuscript.

Funding

This research received no specific grant from any funding agency in the public, commercial or not-for-profit sectors.

Competing interests

The authors declare no potential conflicts of interest with respect to the research, authorship, and/or publication of this article.

References

1. United Nations Department of Economic and Social Affairs. Contraceptive Use by Method 2019.Data Booklet. 2019 [cited 24 Jan 2021]. Available: https://www.un.org/development/desa/pd/sites/www.un.org.development.desa.pd/files/files/documents/2020/Jan/un_2019_contraceptiveusebymethod_databooklet.pdf
2. Khialani D, Jones ME, Szépligeti SK, Ording AG, Ehrenstein V, Petersen I, et al. Combined hormonal contraceptive use in Europe before and after the European Commission mandated changes in product information. Contracept X. 2020;2: 100018. doi:10.1016/j.conx.2020.100018
3. Johnson S, Pion C, Jennings V. Current methods and attitudes of women towards contraception in Europe and America. Reprod Health. 2013;10: 7. doi:10.1186/1742-4755-10-7

4. Dhont M. History of oral contraception. Eur J Contracept Reprod Health Care. 2010;15 Suppl 2: S12-8. doi:10.3109/13625187.2010.513071

5. Burrows LJ, Basha M, Goldstein AT. The effects of hormonal contraceptives on female sexuality: a review. J Sex Med. 2012;9: 2213–2223. doi:10.1111/j.1743-6109.2012.02848.x

6. Hertel J, König J, Homuth G, Van der Auwera S, Wittfeld K, Pietzner M, et al. Evidence for Stress-like Alterations in the HPA-Axis in Women Taking Oral Contraceptives. Sci Rep. 2017;7: 14111. doi:10.1038/s41598-017-13927-7

7. Sitruk-Ware R. Hormonal contraception and thrombosis. Fertil Steril. 2016;106: 1289–1294. doi:10.1016/j.fertnstert.2016.08.039

8. European Medicines Agency. Combined hormonal contraceptives. 2019 [cited 16 Dec 2019]. Available: https://www.ema.europa.eu/en/find-medicine/human-medicines/referrals/combined-hormonal-contraceptives

9. Skovlund CW, Mørch LS, Kessing LV, Lidegaard Ø. Association of Hormonal Contraception With Depression. JAMA Psychiatry. 2016;73: 1154–1162. doi:10.1001/jamapsychiatry.2016.2387

10. Buggio L, Barbara G, Facchin F, Ghezzi L, Dridi D, Vercellini P. The influence of hormonal contraception on depression and female sexuality: a narrative review of the literature. Gynecol Endocrinol. 2022;38: 193–201. doi:10.1080/09513590.2021.2016693

11. Boeschen D, Günther J, Chytrek D, Schoch G-G, Glaeske G, Technikerkrankenkasse. Pillenreport. Ein Statusbericht zu oralen Kontrazeptiva. 2015. Available: https://www.tk.de/resource/blob/2043476/f68a7108f6cdeae1a58e438d312e3ac6/studienband-pillenreport-2015-data.pdf

12. Alves P, Petersen I, Stevenson F. Searching for Information on the Risks of Combined Hormonal Contraceptives on the Internet: A Qualitative Study Across Six European Countries. J Med Internet Res. 2019;21: e10810. doi:10.2196/10810

13. Mitra A. Blood clots, COVID-19 vaccines and the contraceptive pill: are we heading for a repeat of the 1995 pill scare? BMJ Sex Reprod Heal. 2021;47: 303–304. doi:10.1136/bmjsrh-2021-201219

14. Lackie E, Fairchild A. The birth control pill, thromboembolic disease, science and the media: a historical review of the relationship. Contraception. 2016;94: 295–302. doi:10.1016/j.contraception.2016.06.009

15. Foran T. Contraception and the media: lessons past, present and future. Eur J Contracept Reprod Heal Care. 2019;24: 80–82. doi:10.1080/13625187.2018.1563067

16. Spitzer WO. The 1995 pill scare revisited: anatomy of a non-epidemic. Hum Reprod. 1997;12: 2347–2357. doi:10.1093/humrep/12.11.2347

17. Goodyear-Smith F, Arroll B. Termination of pregnancy following panic-stopping of oral contraceptives. Contraception. 2002;66: 163–7.

18. Skjeldestad F. Increased number of induced abortions in Norway after media coverage of adverse vascular events from the use of third generation oral contraceptives. Contraception. 1997;55: 11–4.

19. ntv. Neue Anti-Baby-Pillen Pillenreport warnt vor Thromboserisiko. In: 2015 [Internet]. 2015. Available: https://www.n-tv.de/wissen/Pillenreport-warnt-vor-Thromboserisiko-article16108041.html

20. t-online. Thrombose durch Antibabypille Studie: Neue Generation der Pille birgt Risiken. In: 2015 [Internet]. 2015. Available: https://www.t-online.de/gesundheit/sexualitaet/verhuetung/id_75726592/pillenreport-2015-neue-antibabypille-birgt-risiken.html

21. Focus online. TK PillenreportNeue Antibabypillen haben erhöhtes Thromboserisiko. In: 2015. 2015.

22. Döring N. Mehr Frust als Lust? Die Antibabypille in Sozialen Medien. Medien + erziehung zeitschrift für Medien. 2021;65: 27–34.

23. Bitzer J. Hormonal contraception and depression: another Pill scandal? Eur J Contracept Reprod Heal Care. 2017;22: 1–2. doi:10.1080/13625187.2016.1269163

24. Skovlund CW, Morch LS, Kessing LV, Lange T, Lidegaard O. Association of Hormonal Contraception With Suicide Attempts and Suicides. Am J Psychiatry. 2018;175: 336–342. doi:10.1176/appi.ajp.2017.17060616

25. Kissling EA. No Justice, No Pill/Know (Reproductive) Justice, Know the Pill. Women's Reprod Heal. 2016;3: 109–112. doi:10.1080/23293691.2016.1196085
26. Maas C. #MyPillStory. Frust mit Nebenwirkungen der Pille. Tübingen; 2016.
27. Leask J, Hooker C, King C. Media coverage of health issues and how to work more effectively with journalists: a qualitative study. BMC Public Health. 2010;10: 535. doi:10.1186/1471-2458-10-535
28. Vieth SJ, Hartmann-Boyce J, Maass N, Jani A. Survey of young women's state of knowledge and perceptions about oral contraceptives in Germany. AJOG Glob Reports. 2022;2: 100119. doi:10.1016/j.xagr.2022.100119
29. Broussard K, Becker A. Self-removal of long-acting reversible contraception: A content analysis of YouTube videos. Contraception. 2021;104: 654–658. doi:10.1016/j.contraception.2021.08.002
30. Paul J, Boraas CM, Duvet M, Chang JC. YouTube and the single-rod contraceptive implant: a content analysis. J Fam Plan Reprod Heal Care. 2017;43: 195–200. doi:10.1136/jfprhc-2016-101593
31. Kim JH, Kim HK. Content and quality of YouTube regarding women's health: a scoping review. Korean J Women Heal Nurs. 2023;29: 179–189. doi:10.4069/kjwhn.2023.08.19
32. Pfender EJ, Devlin MM. What Do Social Media Influencers Say About Birth Control? A Content Analysis of YouTube Vlogs About Birth Control. Health Commun. 2023;38: 3336–3345. doi:10.1080/10410236.2022.2149091
33. Wu J, Trahair E, Happ M, Swartz J. TikTok, #IUD, and User Experience With Intrauterine Devices Reported on Social Media. Obstet Gynecol. 2023;141: 215–217. doi:10.1097/AOG.0000000000005027
34. Latack KR, Patel J, Moog D, Spencer D, Nguyen BT. Withdrawal method inquiries and user experiences: An analysis of content posted on 4 gendered forums on Reddit. Contraception. 2021;104: 170–175. doi:10.1016/j.contraception.2021.04.005
35. Döring N, Lehmann S, Schumann-Doermer C. Verhütung auf YouTube, Instagram und TikTok. Bundesgesundheitsblatt - Gesundheitsforsch - Gesundheitsschutz. 2023. doi:10.1007/s00103-

023-03698-0

36. Gomes Alves P, Petersen I, Stevenson F. Searching for Information on the Risks of Combined Hormonal Contraceptives on the Internet: A Qualitative Study Across Six European Countries. J Med Internet Res. 2019;21: e10810. doi:10.2196/10810

37. Döring N. Die Bedeutung von Videoplattformen für die Gesundheitskommunikation. Handbuch der Gesundheitskommunikation. Wiesbaden: Springer Fachmedien Wiesbaden; 2019. pp. 171–183. doi:10.1007/978-3-658-10727-7_14

38. Simmons RG, Baayd J, Waters M, Diener Z, Turok DK, Sanders JN. Assessing contraceptive use as a continuum: outcomes of a qualitative assessment of the contraceptive journey. Reprod Health. 2023;20: 33. doi:10.1186/s12978-023-01573-4

39. Gnoth C, Frank-Herrmann P, Schmoll A, Godehardt E, Freundl G. Cycle characteristics after discontinuation of oral contraceptives. Gynecol Endocrinol. 2002;16: 307–17. Available: http://www.ncbi.nlm.nih.gov/pubmed/12396560

40. Yland JJ, Bresnick KA, Hatch EE, Wesselink AK, Mikkelsen EM, Rothman KJ, et al. Pregravid contraceptive use and fecundability: prospective cohort study. BMJ. 2020; m3966. doi:10.1136/bmj.m3966

41. Davis AR, Kroll R, Soltes B, Zhang N, Grubb GS, Constantine GD. Occurrence of menses or pregnancy after cessation of a continuous oral contraceptive. Fertil Steril. 2008;89: 1059–1063. doi:10.1016/j.fertnstert.2007.05.012

42. Girum T, Wasie A. Return of fertility after discontinuation of contraception: a systematic review and meta-analysis. Contracept Reprod Med. 2018;3: 9. doi:10.1186/s40834-018-0064-y

43. Nassaralla CL, Stanford JB, Daly KD, Schneider M, Schliep KC, Fehring RJ. Characteristics of the Menstrual Cycle After Discontinuation of Oral Contraceptives. J Women's Heal. 2011;20: 169–177. doi:10.1089/jwh.2010.2001

44. Inoue K, Barratt A, Richters J. Does research into contraceptive method discontinuation address women's own reasons? A critical review. J Fam Plan Reprod Heal Care. 2015;41: 292–299. doi:10.1136/jfprhc-2014-100976

45. Kissling EA. What Does Not Kill You Makes You Stronger: Young Women's Online

Conversations about Quitting the Pill. Reframing Reproduction. London: Palgrave Macmillan UK; 2014. pp. 236–250. doi:10.1057/9781137267139_15

46. Basch CH, Menafro A, Mongiovi J, Hillyer GC, Basch CE. A Content Analysis of YouTube™ Videos Related to Prostate Cancer. Am J Mens Health. 2017;11: 154–157. doi:10.1177/1557988316671459

47. Ekrami O, Claes P, White JD, Zaidi AA, Shriver MD, Van Dongen S. Measuring asymmetry from high-density 3D surface scans: An application to human faces. PLoS One. 2018;13. doi:10.1371/journal.pone.0207895

48. Schoonenboom J, Johnson RB. How to Construct a Mixed Methods Research Design. Kolner Z Soz Sozpsychol. 2017/07/05. 2017;69: 107–131. doi:10.1007/s11577-017-0454-1

49. Devendorf A, Bender A, Rottenberg J. Depression presentations, stigma, and mental health literacy: A critical review and YouTube content analysis. Clin Psychol Rev. 2020;78: 101843. doi:10.1016/j.cpr.2020.101843

50. Donzelli G, Palomba G, Federigi I, Aquino F, Cioni L, Verani M, et al. Misinformation on vaccination: A quantitative analysis of YouTube videos. Hum Vaccin Immunother. 2018;14: 1654–1659. doi:10.1080/21645515.2018.1454572

51. Francis JJ, Johnston M, Robertson C, Glidewell L, Entwistle V, Eccles MP, et al. What is an adequate sample size? Operationalising data saturation for theory-based interview studies. Psychol Health. 2010;25: 1229–1245. doi:10.1080/08870440903194015

52. Krippendorff K. Content Analysis: An Introduction to Its Methodology. 2455 Teller Road, Thousand Oaks California 91320: SAGE Publications, Inc.; 2019. doi:10.4135/9781071878781

53. Mayring P. Qualitative Inhaltsanalyse. Grundlagen und Techniken. Weinheim: Beltz Verlagsgrupp; 2015.

54. ADM Arbeitskreis Deutscher Markt- und Sozialforschungsinstitute e.V., Arbeitsgemeinschaft Sozialwissen- schaftlicher Institute e.V. (ASI), BVM Berufsverband Deutscher Markt- und Sozialforscher e.V., Deutsche Gesellschaft für Online Forschung e.V. (DGOF). Richtlinie für Untersuchungen in den und mittels der Sozialen Medien (Soziale Medien Richtlinie). 2021 [cited 3 Feb 2023]. Available: https://www.dgof.de/wp-content/uploads/2021/03/RL-Soziale-Medien-

neu-2021-3.3.2021.pdf

55. Fuchs A, Matonóg A, Sieradzka P, Pilarska J, Hauzer A, Czech I, et al. Anti-androgenic therapy in young patients and its impact on intensity of hirsutism, acne, menstrual pain intensity and sexuality — a preliminary study. Ginekol Pol. 2019;90: 520–526. doi:10.5603/GP.2019.0091

56. Lewandowski SK, Duttge G, Meyer T. Quality of life and mental health in adolescent users of oral contraceptives. Results from the nationwide, representative German Health Interview and Examination Survey for Children and Adolescents (KiGGS). Qual Life Res. 2020;29: 2209–2218. doi:10.1007/s11136-020-02456-y

57. de Castro Coelho F, Barros C. The Potential of Hormonal Contraception to Influence Female Sexuality. Int J Reprod Med. 2019;2019: 9701384. doi:10.1155/2019/9701384

58. Fruzzetti F, Perini D, Fornaciari L, Russo M, Bucci F, Gadducci A. Discontinuation of modern hormonal contraceptives: an Italian survey. Eur J Contracept Reprod Heal Care. 2016;21: 449–454. doi:10.1080/13625187.2016.1234598

59. Grover SR. Gynaecology problems in puberty. Best Pract Res Clin Endocrinol Metab. 2019;33: 101286. doi:10.1016/j.beem.2019.06.002

60. Tracy EE. Contraception. Obstet Gynecol Clin North Am. 2017;44: 143–158. doi:10.1016/j.ogc.2017.02.001

61. Gruber DM. Hormonelle Kontrazeption bei Jugendlichen < 18 Jahren. J für Gynäkologische Endokrinol. 2021;31: 25–28. doi:10.1007/s41974-020-00158-4

62. Both S, Lew-Starowicz M, Luria M, Sartorius G, Maseroli E, Tripodi F, et al. Hormonal Contraception and Female Sexuality: Position Statements from the European Society of Sexual Medicine (ESSM). J Sex Med. 2019;16: 1681–1695. doi:10.1016/j.jsxm.2019.08.005

63. Jenapharm GmbH & Co. KG. Gebrauchsinformation: Information für Anwender. Maxim0,03 mg/2 mg, überzogene Tablette Ethinylestradiol/ Dienogest. Jena. In: 2018 [Internet]. [cited 4 Dec 2021]. Available: https://www.meine-gesundheit.de/medikamente/beipackzettel_Maxim-ueberzogene-Tabletten.pdf/b4f11697-e012-4a5d-b860-76a6529a8f07

64. eurostat. Fertility indicators. 2021. Available: https://ec.europa.eu/eurostat/databrowser/bookmark/bf4fd992-7565-4f68-a499-

a11b487fbb18?lang=en
65. Helfert S, Warschburger P. The face of appearance-related social pressure: gender, age and body mass variations in peer and parental pressure during adolescence. Child Adolesc Psychiatry Ment Health. 2013;7: 16. doi:10.1186/1753-2000-7-16
66. Fardouly J, Pinkus RT, Vartanian LR. The impact of appearance comparisons made through social media, traditional media, and in person in women's everyday lives. Body Image. 2017;20: 31–39. doi:10.1016/j.bodyim.2016.11.002
67. Gattario KH, Frisén A. From negative to positive body image: Men's and women's journeys from early adolescence to emerging adulthood. Body Image. 2019;28: 53–65. doi:10.1016/j.bodyim.2018.12.002
68. Samuels D V., Rosenthal R, Lin R, Chaudhari S, Natsuaki MN. Acne vulgaris and risk of depression and anxiety: A meta-analytic review. J Am Acad Dermatol. 2020;83: 532–541. doi:10.1016/j.jaad.2020.02.040
69. Barnhart KT, Schreiber CA. Return to fertility following discontinuation of oral contraceptives. Fertil Steril. 2009;91: 659–663. doi:10.1016/j.fertnstert.2009.01.003
70. Kristjánsdóttir J, Sundelin C, Naessen T. Health-related quality of life in young women starting hormonal contraception: a pilot study. Eur J Contracept Reprod Heal Care. 2018;23: 171–178. doi:10.1080/13625187.2018.1455179
71. Mansour D, Inki P, Gemzell-Danielsson K. Efficacy of contraceptive methods: A review of the literature. Eur J Contracept Reprod Heal Care. 2010;15: 4–16. doi:10.3109/13625180903427675
72. Frank-Herrmann P, Heil J, Gnoth C, Toledo E, Baur S, Pyper C, et al. The effectiveness of a fertility awareness based method to avoid pregnancy in relation to a couple's sexual behaviour during the fertile time: a prospective longitudinal study. Hum Reprod. 2007;22: 1310–1319. doi:10.1093/humrep/dem003
73. Freundl G, Frank-Herrmann P, Brown S, Blackwell L. A new method to detect significant basal body temperature changes during a woman's menstrual cycle. Eur J Contracept Reprod Heal Care. 2014;19: 392–400. doi:10.3109/13625187.2014.948612
74. Minnis AM, Mavedzenge SN, Luecke E, Dehlendorf C. Provider Counseling to Young Women

Seeking Family Planning Services. Perspect Sex Reprod Health. 2014;46: 223–231. doi:10.1363/46e1414

75. Goldhammer DL, Fraser C, Wigginton B, Harris ML, Bateson D, Loxton D, et al. What do young Australian women want (when talking to doctors about contraception)? BMC Fam Pract. 2017;18: 35. doi:10.1186/s12875-017-0616-2

76. Dixon SC, Herbert DL, Loxton D, Lucke JC. 'As many options as there are, there are just not enough for me': Contraceptive use and barriers to access among Australian women. Eur J Contracept Reprod Heal Care. 2014;19: 340–351. doi:10.3109/13625187.2014.919380

77. Dehlendorf C, Krajewski C, Borrero S. Contraceptive Counseling. Clin Obstet Gynecol. 2014;57: 659–673. doi:10.1097/GRF.0000000000000059

78. Branden P. Contraceptive choice and patient compliance The health care provider's challenge. J Nurse Midwifery. 1998;43: 471–482. doi:10.1016/S0091-2182(98)00058-5

79. de Irala J, Osorio A, Carlos S, Lopez-del Burgo C. Choice of birth control methods among European women and the role of partners and providers. Contraception. 2011;84: 558–564. doi:10.1016/j.contraception.2011.04.004

80. Gallagher KM, Updegraff JA. Erratum to: Health Message Framing Effects on Attitudes, Intentions, and Behaviors: A Meta-analytic Review. Ann Behav Med. 2013;46: 127–127. doi:10.1007/s12160-012-9446-6

Supporting information

S1. German search terms

S2. Translated codebook

S3. Qualitative category system

Click here to access/download
Supporting Information
S1_Search Strategy.docx

Click here to access/download
Supporting Information
S2_QUANCodebook.docx

Click here to access/download
Supporting Information
S3_QUALCoding Scheme.docx

Click here to access/download
Supporting Information
Supplementary Table 1 .docx
