# Supplementary material for: YouTube and the implementation and discontinuation of the oral contraceptive pill: A mixed-method content analysis
Source: PLoS One. 2024 May 24;19(5):e0302316. doi: 10.1371/journal.pone.0302316 (PMC11125465; doi:10.1371/journal.pone.0302316)
Supplement: Attachment — Submitted filename: Response to reviewers.docx [file pone.0302316.s006.docx]

**A point-by-point response to reviewers**

Dear Dr Deidre Pretorius, dear reviewers,

Thank you very much for your thoughtful comments and for the opportunity to resubmit our article. We are grateful that you are considering publishing it in *PLosOne*.

Please find below our responses to your comments and the reviewers’ comments. Our responses are highlighted *in italics*. We have marked changes to the previous version in red font in the marked-up copy of the manuscript.

We are looking forward to your feedback.

With kind regards,

Jana Niemann, Lea Wicherski, Lisa Glaum, Liane Schenk, Getraud Stadler, Matthias Richter

**Journal Requirements**

1. Please ensure that your manuscript meets PLOS ONE's style requirements, including those for file naming.

*Authors: We have adjusted the manuscript format to PLOS ONE’s style requirements.*

2. In your Methods section, please include additional information about your dataset and ensure that you have included a statement specifying whether the collection and analysis method complied with the terms and conditions for the source of the data.

*Authors: We have added the following statement to the end of the ethics section: “The collection and analysis method complied with the terms and conditions for the source of the data.”*

3. We note that your Data Availability Statement is currently as follows: [All relevant data are within the manuscript and its Supporting Information files.]

Please confirm at this time whether or not your submission contains all raw data required to replicate the results of your study. Authors must share the “minimal data set” for their submission. PLOS defines the minimal data set to consist of the data required to replicate all study findings reported in the article, as well as related metadata and methods (https://journals.plos.org/plosone/s/data-availability#loc-minimal-data-set-definition).

For example, authors should submit the following data:

- The values behind the means, standard deviations and other measures reported;

- The values used to build graphs;

- The points extracted from images for analysis.

Authors do not need to submit their entire data set if only a portion of the data was used in the reported study.

If your submission does not contain these data, please either upload them as Supporting Information files or deposit them to a stable, public repository and provide us with the relevant URLs, DOIs, or accession numbers. For a list of recommended repositories, please see https://journals.plos.org/plosone/s/recommended-repositories.

If there are ethical or legal restrictions on sharing a de-identified data set, please explain them in detail (e.g., data contain potentially sensitive information, data are owned by a third-party organization, etc.) and who has imposed them (e.g., an ethics committee). Please also provide contact information for a data access committee, ethics committee, or other institutional body to which data requests may be sent. If data are owned by a third party, please indicate how others may request data access.

*Authors: With this revision, we have added the raw data of the quantitative data to our OSF project and added the following sentence:*

*“The study follows the Open Science movement, i.e. the pre-registration, and all quantitative data are stored on the Open Science Foundation server (https://osf.io/fekdh/).”*

*For the qualitative research, we would not feel comfortable uploading the raw transcripts with the manuscript. We want to provide some privacy to the participants in the study.*

4. Please review your reference list to ensure that it is complete and correct. If you have cited papers that have been retracted, please include the rationale for doing so in the manuscript text, or remove these references and replace them with relevant current references. Any changes to the reference list should be mentioned in the rebuttal letter that accompanies your revised manuscript. If you need to cite a retracted article, indicate the article’s retracted status in the References list and also include a citation and full reference for the retraction notice.

*Authors: We have gone through all references of the revised manuscript. We have found no retracted articles. We have added the following references to accompany the revision:*

- *40. Ross LJ. Reproductive Justice as Intersectional Feminist Activism. Souls. 2017;19: 286–314. doi:10.1080/10999949.2017.1389634*
- *41. Castle S, Askew I, Population Council. CONTRACEPTIVE DISCONTINUATION: REASONS, CHALLENGES, AND SOLUTIONS. 2015. Available:* [*https://popdesenvolvimento.org/images/imprensa/FP2020_ContraceptiveDiscontinuation_SinglePageRevise_12.16.15.pdf*](https://popdesenvolvimento.org/images/imprensa/FP2020_ContraceptiveDiscontinuation_SinglePageRevise_12.16.15.pdf)
- *60.Patterson AN. YouTube Generated Video Clips as Qualitative Research Data: One Researcher’s Reflections on the Process. Qual Inq. 2018;24: 759–767. doi:10.1177/1077800418788107*

*We have deleted one citation from the text, as we cited it incorrectly:*

- *Ekrami O, Claes P, White JD, Zaidi AA, Shriver MD, Van Dongen S. Measuring asymmetry from high-density 3D surface scans: An application to human faces. PLoS One. 2018;13. doi:10.1371/journal.pone.0207895*

*We have added the DOI to the following citation:*

- *43. Gnoth C, Frank-Herrmann P, Schmoll A, Godehardt E, Freundl G. Cycle characteristics after discontinuation of oral contraceptives. Gynecol Endocrinol. 2002;16: 307–317. doi:10.1080/gye.16.4.307.317*

*We have added the URL to the following citation:*

- *64. Hardon A, Harries J. Towards an Anthropology of contraception: on the pill, control and embodiment. AM Riv Della Soc Ital Di Antropol Medica. 2001;6: 211–226. Available:* [*https://www.amantropologiamedica.unipg.it/index.php/am/article/view/119/112*](https://www.amantropologiamedica.unipg.it/index.php/am/article/view/119/112)

*We have adapted the title in the following citation:*

- *67. Tracy EE. Contraception: Menarche to Menopause. Obstet Gynecol Clin North Am. 2017;44: 143–158. doi:10.1016/j.ogc.2017.02.001*

*Finally, we have adapted the following citation:*

- *87. Gallagher KM, Updegraff JA. Health Message Framing Effects on Attitudes, Intentions, and Behavior: A Meta-analytic Review. Ann Behav Med. 2012;43: 101–116. doi:10.1007/s12160-011-9308-7*

**Editor**

Interesting and innovative research however, it needs serious technical attention.

*Authors: Thank you for your feedback. We have gone through the entire manuscript with an eye for technical errors. We also hired a professional to proofread the manuscript.*

**Reviewer 1 - Janine White**

This was a very interesting study exploring OCP discontinuation using social media (Youtube) and I enjoyed reading it. This study demonstrates the utility of using Youtube as an acceptable way to explore the lived experiences of women and their choices on OCP, particularly discontinuation. I have a few comments which I hope will strengthen the paper:

Introduction

*Authors: Dear Janine White, thank you for your encouraging remarks and for challenging us to improve the paper. We detail below whether and how we addressed your comments.*

- p.4 line 37 remove the brackets from mainstream. It is an important concept that described the locality of Youtube in social media and should not be in brackets.

*Authors: We have adjusted the text passage accordingly.*

- p.4 line 40 Pill-scare - perhaps consider adding the word phenomenon to bring across that it was an observed situation that was in question. Also, add where this took place - was it a global phenomenon? Or mostly in US or Europe = HICs?

*Authors: Thank you very much. We changed the wording of the sentences to “An important example is the “pill scare” phenomenon of 1995 [16–18], focusing on high-income countries.” (see lines 41-42).*

- p.4 line 43 which countries did those studies on unintended pregnancies and abortion take place?

*Authors: We have added the countries to the references.*

- p.4 line 44 instead of beginning the sentence with 'however', consider using 'while'

*Authors: We have adjusted the text passage accordingly.*

- p.4 line 45 In Germany, this stance was also taken up... (add the word stance). Also, expand a little more on the pill report for context.

*Authors: We have added “stance” and more information on the pill report: “This stance was also taken up in the OCP report [11] in Germany in 2015, which lacked scientific rigor [19]. The authors concluded there was a higher risk of thrombosis with the more mod-ern 3rd and 4th generation OCPs. This was further disseminated by the media [20–22].” (lines 47-49)*

- p.4 line 47 ...the media focused on the findings of increased risk of depression...(add the word findings for better coherence of this sentence)
- *Authors: We have added the word “findings” accordingly.*
- p.4 line 48 consider change the word context to community

*Authors: We have exchanged the word accordingly.*

- p.4 line 49 are the weaknesses that the findings could not be generalised?

*Authors: We have added the following sentence to give examples of the criticism: “Bitzer (2017), for example, criticized the lack of sensitivity analysis and questioned the biological plausibility [24].” (lines 52-53)*

- p.5 line 55 rephrase this sentence for better clarity

*Authors: We have reworded the sentence and hope that it is now clearer: “In summary, the media discussion of OCPs has underrepresented studies that do not show cor-relations with adverse side effects and have often failed to distinguish between absolute and rela-tive risks [15,23].” (lines 59-60)*

- p.5 line 59 remove the brackets from hormonal

*Authors: We have removed the brackets.*

- p.5 line 69 and 'on these sites' to bring across that the shortcoming were related to these sites

*Authors: We have added the word “on these sites” accordingly.*

- p.5 line 76 Add an in-text citation for the S&R Justice Framework; also expand more on this justice lens and it's relevance to your study

*Authors: We added an in-text citation and expanded on the justice lens regarding contraceptive agency and contraceptive choice: “Informed choice regarding contraceptive methods is an important component of the Sexual and Reproductive Justice Framework [26,40]. Knowledge and information can contribute to achieving justice through increased reproductive agency and contraceptive choice [26].” (lines 79-81)*

- p.6 line 80 comma after reason. In text citation for that sentence as it is a definition (or it is the way the researchers have defined it in the study of drawn from a definition in the literature?). Please indicate what the German study was a bout, In a German study on...

*Authors: We added the comma to the sentence and indicated what the German study was about. We also added a direct quote from a similar definition of discontinuation with a reference: “The OCP can be defined as “starting contraceptive use and then stopping for any reason while still at risk of an unintended pregnancy” [41]. A German study on the knowledge and perceptions about OCPs among young women showed that 64.5 % (n = 1480) of those who used OCPs at some point discontinued the method [29].” (lines 84-87)*

- Throughout the document please ensure that your in-text citations are in line with the journal requirements - usually at the end of the sentence, rather than for example next to the authors surname at the beginning of the sentence. This may just be a difference in our styles so ensure it meets the journal requirements.

*Authors: We have adjusted the citations accordingly.*

- p.6 line 84 relationships...with? Is it intimate/family/friends?

*Authors: We have added examples for the possible relationships.*

- p.6 line 94 mostly positive = in what kind of ways? Agreement? or shared experiences of discontinuation

*Authors: We have added that the reactions were “very positive with many views and likes, very few dislikes and lots of friendly comments.” (lines 100-101)*

- p.6 line 103 live video and recordings?

*Authors: We have deleted the word “live” from the sentence”*

- p.7 lines 109 - 116 perhaps consider an overall research question or aim, with these RQs reframed as objectives? It will make it easier to begin that opening paragraph in the discussion a lot more succinct and coherent. See my comment on that later.

*Authors: We have rewritten the objectives and research questions as you suggested. We hope that the paragraph and the beginning of the discussion are clearer now: “The overall objective of this content analysis was to investigate the personal physiological and psychological changes and lived experiences of German-speaking YouTubers after initiation and discontinuation of OCP treatment. We, therefore, aim to examine*

- *the reasons for starting and discontinuing the use of the OCP,*
- *to document any side effects experienced during and after use, and*
- *how the women describe their history with the OCP.” (lines 114-119)*

METHODS

- P.7 line 119 pre-registered - what does pre-reg involve? Was that of the study of the video?

*Authors: We have clarified that we registered a protocol for this study and restructured the beginning of the paragraph as follows: “The study follows the Open Science movement, i.e. the pre-registration, and all data are stored on the Open Science Foundation server (*[*https://osf.io/fekdh/*](https://osf.io/fekdh/)*). It was designed as an (almost) simultaneous exploratory mixed-methods content analysis [52]” (lines 122-124).*

- p.7 line 121 were findings interpreted together? Where di the mixing take place? In the abstract the researchers state that this was a concurrent explanatory study - this must come through clearly in the design section of this paper.

*Authors: We have rearranged the paragraph of the study design and added that: “The qualitative and quantitative research strands were initially analyzed separately and then brought together for the interpretation and presentation of the results.” (lines 127-128).*

- p.7 126-127 keep the date format consistent. Also explain why the browser and cookies history were cleared & how this links to algorithms and what shows up in your search results/feed?

*Authors: We’ve changed the date format and explained why we cleared the browser and cookies history: “YouTube was initially searched from July 20 to 23, 2021. An updated search was performed on May 5, 2023. The internet browser and cookies history were cleared before the search to avoid biases from our laptop.” (lines 130-132)*

- p.8 line 135 = what was the relevance filter?

*Authors: We have added the relevance filter (=year) to the sentence*

- p.8 line 140 If repeated

*Authors: We considered your suggestion and adapted the sentence.*

- p.9 the flow chart should have arrows illustrating how the decisions took place

*Authors: We are not sure what you are referring to. We have based this flowchart on the PRISMA flowchart to illustrate the decision process. The flowchart includes arrows to indicate direction and how the boxes are linked.*

- p.10 line 188 registration of?

*Authors: We have deleted the “registration of” from the sentence.*

- p.11 line 199 Who did the transcribing?

*Authors: We have added the authors to the sentence.*

- How could you assess that they are adults? Could this be a limitation?

*Authors: Yes, this is indeed a limitation. For more than half of the participants, we know the age at which they stopped. For the rest of the enrolled participants, we only assumed they were adults based on appearance and previous upload history.*

*We have added this limitation to the strengths and limitations section.: “Furthermore, for a large proportion of our study participants, we had to assume from looks and past upload history that they were adults.” (lines 464-465)*

- p.11 line 200 where did you obtain ethical clearance to conduct this study? I understand that informed consent wasn't a requirement (as per lines 202 - 203). Also, please expand on the ethical appropriateness of conducting the study without informed consent from the Youtubers.

*Authors: We have added our ethical approval for the study. In addition, we have further considered the ethical appropriateness of our data: “The ethical approval for this study has been granted by the Ethical Review Committee of the Medical Faculty at Martin Luther University Halle-Wittenberg.” (lines 211- 212).*

*And have extended a little bit on the ethical appropriateness of conducting the study: “Before a person uploads a video on YouTube, they have to consent that their data can be reused through third parties [59]. Therefore, a certain level of awareness can be assumed [60]. We are aware that this requires a certain level of maturity. Therefore, we decided to only include adult YouTubers who are aware of their publicity (e.g. through follower numbers) and the publicly available works.”*

- p.11 line 208 what do you mean by different media?

*Authors: We have added that: “The personal data (e.g. title, YouTube channel name) were separated from the factual data (e.g. time of posting, views) and stored on different media (USB key).”*

RESULTS

- p.12 line 226 facial skin = facial skin impurities? please ensure this is consistent throughout to illustrate that it is the impurities that became a problem rather than just facial skin.

*Authors: Where appropriate we added “impurities”.*

- p.12 line 229 is it about easing menstrual impurities?

*Authors: Yes, we have added “easing” to the menstrual cramps.*

- p.12 lines 235 - 236 Is it about irregular periods or a decrease in relation to heavy bleeding?

*Authors: This was related to heavy bleeding.*

- p.1 line 246 add an example so it is consistent with the previous results

*Authors: We have added the examples accordingly.*

- p.13 247 mention % in the table = frequency?

*Authors: We have exchanged the word “mention” through “frequency”*

- p.14 line 248 double nomination = overlap?

*Authors: We have exchanged the words “double nomination” through “overlap”*

- p.15 line 268 what is the meaning of extreme in this context of libido?

*Authors: We have added “intense” instead of extreme as we feel that it describes the return of libido more appropriately.*

- p.15 line 271 restructure sentence for better coherence = 32 women experienced psychological changes..

*Authors: We have adjusted the sentence accordingly.*

- p.15 line 272 An increase in facial skin blemishes were..

*Authors: We have adjusted the sentence accordingly.*

- p.15 line 275 remove exclamation point (!)

*Authors: Thank you, we have removed the exclamation point.*

- p.16 line 293 mention = frequency

*Authors: We have exchanged the word “mention” through “frequency”*

- p.20 line 315 very interesting please expand a bit more here on "not an issue between a man and woman"

*Authors: We have added the direct quote from the transcript and expanded more on that: “The general cost of contraception as a female burden was also mentioned. P130 described that:*

*“Of course, it costs money, and you shouldn’t forget that either. It’s also really absurd that we women have to pay for it and the men don’t. Not because of men and women, but I think the health insurance should pay for it. I can’t even think about paying that much a month. And I never go to the doctor. Never. I never have anything. I don’t even know what for.”*

*What is interesting here is that they see the financial burden as something that should be negotiated and shared between men and women”. (lines 333-336)*

DISCUSSION

- p.21 lines 339 - 354 My suggestion is that you start with the overall RQ (I suggested earlier) or aim and then highlight the key findings from the study. Be more coherent in this opening paragraph of the discussion. At the moment, it is written so that it overlaps with the results section.

*Authors: We have rewritten the paragraph to improve its coherence:* “*This analysis demonstrates that bodily experiences and the body itself are significant components in OCP autobiographies, from initiation to the period after discontinuation. The primary reasons for initiation are to address facial skin impurities, use as a contraceptive method, and to alleviate painful menstrual cramps. Common side effects experienced during the use of OCPs include mood swings, weight gain, headaches, depressed mood, decreased libido, water retention, and migraines. Women have described these effects as burdensome, despite the OCPs serving both medical and contraceptive purposes. Women predominantly discontinue OCPs due to side effects, a desire to cease hormone intake, and increased awareness of related issues. After discontinuation, individuals commonly reported improvements in facial skin, hair, and weight loss, reduced mood swings, increased libido, and a generally positive experience of feeling more connected to their bodies and freer. To the best of the authors’ knowledge, this is one of the few studies on the personal views and experiences of women who have discontinued the OCP. The combination of quantitative description and qualitative analysis in interpreting the results was not only helpful in measuring experiences with the OCP, they showed how these experiences are perceived and lived. The results of this study are consistent with scientific research on the positive and negative side effects [61–63] of OCPs, the reasons for discontinuation [29,48,64], and embodiment [65,66].” (lines 361- 376).*

- p.22 lines 372 The main reported reasons...

*Authors: We have adjusted the sentence accordingly.*

- p.23 line 391 caution in interpretation = why?

*Authors: We have added there is still no consistency in the direction of the association between psychological factors and the contraceptive pill: “Research on these topics should still be interpreted with caution, as there is limited consistency in the direction of the evidence [10]. (lines 412-413).*

- p.23 line 408 in-text citation is missing here and you're citing a German study.

*Authors: We have added the in-text citation.*

- p.24 line 424 ...method choice, particularly discontinuation. Remove brackets.

*Authors: We have removed the brackets.*

- p.24 line 425 add how these choices change over the life course.

*Authors: We have added this with a reference.*

- p.24 line 431 ...information publicly, especially in the context (merge the sentences)

*Authors: We have merged the sentences as you suggested.*

- p.24 line 434 please emphasise where the mixing of methods took place?

Authors: *We have added an emphasis on the mixing of the research strands at the start of the discussion and hope that it is clearer now: “. The combination of quantitative description and qualitative analysis in interpreting the results was not only helpful in measuring experiences with the OCP, they showed how these experiences are perceived and lived.” (lines 372-374)*

**Reviewer 2**

This study was an innovative method for data collection as it avoided ethical considerations as well as labour-intensive participant recruitment.

- It does, however, have some limitations in terms of the validation methods for the qualitative aspect of this study. Because the data is retrospective, iteration was not possible. It was also not possible for triangulation as another method to validate your results. Perhaps self-reflection (reflexivity) should also be considered. These are just aspects to acknowledge. I do not think it disqualifies this study from being published.

*Authors: Thank you very much for these thoughtful comments. We have added the limitations mentioned above to the “strengths and limitations section”: “The study is also limited in terms of validating the qualitative aspect of this study as the data is retrospective, thus, preventing iteration and triangulation as additional validation methods.”. (lines 465-466)*

*In addition, we have added a statement of reflexibility of the two researchers in the qualitative data analysis: “Reflexibility. The two main researchers involved in the qualitative research (JN and LW) have taken and stopped the OCP themselves. Although we were not required to participate in interviews, our lived experiences were part of the knowledge production in this study. Both researchers talked about the process of coding and creating categories throughout the data analysis.” (lines 205-209)*

- There are some typing errors and many grammatical problems. Many sentences are difficult to understand. I would suggest proofreading and language editing to be done by a certified professional before resubmission. I have highlighted a few typing errors and confusion re meaning of terms or sentences, but my role was not to proofread, or language edit this manuscript as other professionals should assist you with it.

*Authors: Thank you very much, the manuscript is now thoroughly checked by professional proofreading.*
